# ﻿A preliminary checklist of the Diptera of Armenia

**DOI:** 10.3897/zookeys.1262.168630

**Published:** 2025-12-08

**Authors:** Meri Arzumanyan, Natela Tarzyan, Björn Rulik, Adrian Pont

**Affiliations:** 1 Research Institute of Biology, Yerevan State University, Yerevan, Armenia; 2 Faculty of Biology, Yerevan State University, Yerevan, Armenia; 3 Zoologisches Forschungsmuseum Alexander Koenig, Bonn, Germany; 4 Oxford University Museum of Natural History, Oxford, UK

**Keywords:** Arthropods, biodiversity, insects, invertebrates, Caucasus

## Abstract

This paper presents the first comprehensive checklist of the Diptera of Armenia, encompassing 53 families and more than 1361 species, including 106 species recorded for the first time in the country. These new records significantly expand the known dipteran fauna of the region, highlighting both the biological richness of Armenia and the substantial gaps that remain in our knowledge of the Diptera of the region. New records are given in the families Anthomyiidae, Muscidae, Scathophagidae and Sciomyzidae. The checklist has been compiled through a synthesis of historical literature, museum collections, and recent field surveys, and will provide an essential reference point for future faunistic, ecological, and conservation studies. Given the ecological sensitivity of many dipteran groups and their utility as bioindicators, this baseline inventory will contribute not only to taxonomy and biogeography but also to broader environmental monitoring efforts within Armenia.

## ﻿Introduction

Armenia is located at the crossroads of the Caucasus ecoregion, which is a unique and rich biodiversity hotspot with complex topography and various climatic zones (Myers et al. 2000; Zazanashvili et al. 2004). Armenia is estimated to have a total animal diversity of 17,000 species including 12,000 species of insects; more than 300 species of animals are presented as endemic to the country (Fayvush 2023). Among insects, Diptera are one of the more poorly studied orders of insects. The earliest works from Armenia cover the family Simuliidae (Terterian 1968), while some pest species were studied in the 1970s (Avetyan and Mirzoyan 1976; Mirzoyan 1977). The few works that included family records from Armenia covered the Caucasus region in general or the entire USSR (Rohdendorf 1937; Paramonov 1940; Richter 1968), and consequently there are very few records from Armenia for most families. Most of the works regarding different families are scattered in different publications and catalogues which necessitates the creation of a united list of all publicly available data for Diptera of Armenia.

The goal of this study was to create a checklist of all known Diptera for Armenia.

## ﻿Materials and methods

Records have been obtained from published research articles, books, and catalogues, and also from on-line catalogues of some individual families. These sources reveal that the sampling of different families of the Armenian Diptera has taken place at various times in the past, according to the specialities of individual investigators. Fundamental to our work has been the Catalogue of Palaearctic Diptera (Soós and Papp 1984–1993). Catalogues and monographs of individual families are mentioned below under the relevant family. Most names have been checked for their availability and validity with the on-line *Systema Dipterorum* (Evenhuis and Pape 2025). The presented format for the checklist is arranged alphabetically within two broad categories of Brachycera and “Nematocera”.

New records in the present checklist will be found in the family Anthomyiidae, based on collections in the Oxford University Museum of Natural History, UK; in the Muscidae, in the Zoological Institute, Russian Academy of Sciences, St Petersburg, Russia; and in the Sciomyzidae and Scathophagidae, in the Natural History Museum, London, UK.

This checklist does not include species that have been recorded simply from the “Caucasus” or from “Transcaucasus”. The species recorded here in this checklist have all been listed at least from “Armenia”, although frequently specific localities have also been mentioned.

## ﻿Results

The number of species in each family of Diptera known from Armenia are given below (Table 1).

**Table 1.** Checklist of the Diptera of Armenia.

N	Family	Number of recorded species	Number of new records
** Brachycera **			
1	Agromyzidae	12	0
2	Anthomyiidae	4	71
3	Anthomyzidae	1	0
4	Asilidae	39	0
5	Bombyliidae	253	0
6	Calliphoridae	28	0
7	Chamaemyiidae	19	0
8	Chloropidae	8	0
9	Conopidae	18	0
10	Curtonotidae	1	0
11	Dolichopodidae	25	0
12	Drosophilidae	2	0
13	Empididae	10	0
14	Ephydridae	16	0
15	Fanniidae	20	0
16	Hybotidae	3	0
17	Lauxaniidae	1	0
18	Micropezidae	1	0
19	Muscidae	184	24
20	Mythicomyiidae	5	0
21	Opomyzidae	2	0
22	Phaeomyiidae	1	0
23	Piophilidae	1	0
24	Platystomatidae	7	0
25	Rhinophoridae	1	0
26	Sarcophagidae	83	0
27	Scathophagidae	2	6
28	Sciomyzidae	11	5
29	Sepsidae	18	0
30	Sphaeroceridae	6	0
31	Stratiomyidae	22	0
32	Tabanidae	36	0
33	Tachinidae	68	0
34	Tephritidae	12	0
35	Therevidae	8	0
36	Ulidiidae	2	0
“**Nematocera**”			
37	Anisopodidae	2	0
38	Bibionidae	4	0
39	Blephariceridae	1	0
40	Cecidomyiidae	126	0
41	Ceratopogonidae	49	0
42	Chironomidae	7	0
43	Culicidae	31	0
44	Dixidae	3	0
45	Keroplatidae	3	0
46	Limoniidae	62	0
47	Mycetophilidae	5	0
48	Pediciidae	5	0
49	Psychodidae	37	0
50	Ptychopteridae	2	0
51	Scatopsidae	3	0
52	Simuliidae	60	0
53	Tipulidae	31	0
	**Total**	**1361**	**106**

The species listed below with “*” are new records for Armenia.

### ﻿Diptera: Brachycera

#### ﻿Agromyzidae

The family Agromyzidae contains many species that are pests of various crops and vegetables, and these have been studied in Armenia by several authors (Avetyan and Mirzoyan 1976; Mirzoyan 1977). Their distribution may be restricted to specific host-plants in specific areas, or may cover large parts or even the whole of Armenia.

1. *Agromyza salicifolii* Collin, 1911

2. *Aulagromyza tridentata* (Loew, 1858)

3. *Cerodontha denticornis* (Panzer, 1806)

4. *Cerodontha incisa* (Meigen, 1830)

5. *Euhexomyza schineri* (Giraud, 1862)

6. *Euhexomyza simplicoides* (Hendel, 1920)

7. *Liriomyza strigata* (Meigen, 1830)

8. *Phytagromyza hendeliana* Hering, 1926

9. *Phytobia elaeagni* Rohdendorf-Holmanová, 1959

10. *Phytomyza atricornis* Meigen, 1838

11. *Phytomyza potentillae* (Kaltenbach, 1864)

12. *Phytomyza xylostei* Goureau, 1851

#### ﻿Anthomyiidae

The list of Anthomyiidae also includes published records of several species that are serious agricultural pests in Armenia, such as *Delia platura*, and that are predators of black flies (Avetyan and Mirzoyan 1976; Ackland and Werner 2006). The list below is based on collections made by ACP in all parts of Armenia which were identified by the late Michael Ackland (UK) and which are now deposited in the Oxford University Museum of Natural History (UK).

1. *Acyglossa atramentaria (Meigen, 1826)

2. *Adia cinerella (Fallén, 1825)

3. *Alliopsis billbergi (Zetterstedt, 1838)

4. *Alliopsis pilitarsis (Stein, 1900)

5. *Alliopsis simulivora Ackland in Ackland & Werner, 2006

6. *Anthomyia liturata (Robineau-Desvoidy, 1830)

7. *Anthomyia pluvialis (Linnaeus, 1758)

8. *Anthomyia procellaris Rondani, 1866

9. *Botanophila discreta (Meigen, 1826)

10. *Botanophila dissecta (Meigen, 1826)

11. *Botanophila extensa Ackland, 2008

12. *Botanophila fugax (Meigen, 1826)

13. *Botanophila intermedia (Zetterstedt, 1860)

14. *Botanophila turcica (Hennig, 1972)

15. *Botanophila varicolor (Meigen, 1826)

16. *Botanophila verticella (Zetterstedt, 1838)

17. *Calythea nigricans (Robineau-Desvoidy, 1830)

18. *Delia angustaeformis (Ringdahl, 1933)

19. *Delia antiqua (Meigen, 1826)

20. *Delia bracata (Rondani, 1866)

21. *Delia cardui (Meigen, 1826)

22. *Delia carduiformis (Schnabl in Schnabl & Dziedzicki, 1911)

23. *Delia coarctata* (Fallén, 1825)

24. *Delia cuneata Tiensuu, 1946

25. *Delia echinata (Séguy, 1923)

26. *Delia giresunensis Hennig, 1974

27. *Delia floralis* (Fallén, 1824)

28. *Delia hirticrura (Rondani, 1871)

29. *Delia lophota (Pandellé, 1900)

30. *Delia majuscula (Pokorny, 1889)

31. *Delia megatricha (Kertész, 1901)

32. *Delia penicilliventris Ackland, 2010

33. *Delia penicillosa Hennig, 1974

34. *Delia platura* (Meigen, 1826)

35. *Delia podagricicauda Xue, 1997

36. *Delia radicum (Linnaeus, 1758)

37. *Enneastigma compressum (Stein, 1916)

38. *Eutrichota inornata (Loew, 1873)

39. *Heterostylodes nominabilis (Collin, 1947)

40. *Heterostylodes obscura (Macquart, 1835)

41. *Hydrophoria linogrisea (Meigen, 1826)

42. *Hydrophoria ruralis (Meigen, 1826)

43. *Hydrophoria silvicola (Robineau-Desvoidy, 1830)

44. *Hylemya nigrimana (Meigen, 1826)

45. *Hylemya partita (Meigen, 1826)

46. *Hylemya urbica (Wulp, 1896)

47. *Hylemya vagans (Panzer, 1798)

48. *Hylemya variata (Fallén, 1823)

49. *Lasiomma seminitidum (Zetterstedt, 1845)

50. *Lasiomma strigilatum (Zetterstedt, 1838)

51. *Leucophora cinerea Robineau-Desvoidy, 1830

52. *Leucophora personata (Collin, 1922)

53. *Leucophora sponsa (Meigen, 1826)

54. *Leucophora unistriata (Zetterstedt, 1838)

55. *Paradelia intersecta (Meigen, 1826)

56. *Paregle audacula (Harris, 1780)

57. *Pegomya bicolor (Wiedemann, 1817)

58. *Pegomya conformis (Fallén, 1825)

59. *Pegomya cunicularia (Rondani, 1866)

60. *Pegomya flavifrons (Walker, 1849)

61. *Pegomya geniculata (Bouché, 1834)

62. *Pegomya hybernae Michelsen, 1988

63. *Pegomya hyoscyami* (Panzer, 1809)

64. *Pegomya meridiana (Villeneuve, 1923)

65. *Pegomya sollennis (Meigen, 1826)

66. *Pegomya ulmaria (Rondani, 1866)

67. *Pegoplata aestiva (Meigen, 1826)

68. *Pegoplata infirma (Meigen, 1826)

69. *Phorbia curvicauda (Zetterstedt, 1845)

70. *Phorbia fumigata (Meigen, 1826)

71. *Phorbia genitalis (Schnabl in Schnabl & Dziedzicki, 1911)

72. *Phorbia lobata (Huckett, 1929)

73. *Phorbia securis* Tiensuu, 1935

74. *Subhylemyia longula (Fallén, 1824)

75. *Zaphne divisa (Meigen, 1826)

#### ﻿Anthomyzidae

Only a single record has been found for this family, in Roháček (2006) where Armenia is included in the distribution of this species.

1. *Anthomyza gracilis* Fallén, 1823

#### ﻿Asilidae

The earliest records for Asilidae are found in the monograph by Richter (1968). Further records are given by Ghahari et al. (2007, 2014) and Mohammadi and Khaghaninia (2016). *Machimus erevanensis* Richter, 1963 and *Satanas gigas* Evermann, 1885 are included in the Red Book of Armenia (2010).

1. *Acnephalum leucostomum* (Engel, 1930)

2. *Acnephalum mixtum* (Loew, 1847)

3. *Anarolius jubatus* Loew, 1844

4. *Cerdistus denticulatus* (Loew, 1849)

5. *Cerdistus manii* Schiner, 1867

6. *Crobilocerus megilliformis* (Loew, 1847)

7. *Didysmachus picipes* (Meigen, 1820)

8. *Dysmachus bilobus* Loew, 1871

9. *Dysmachus dasyproctus* Loew, 1871

10. *Dysmachus stylifer* (Loew, 1854)

11. *Eremisca araratica* Richter, 1966

12. *Eutolmus calopus* (Loew, 1848)

13. *Eutolmus parricida* (Loew, 1848)

14. *Habropogon doriae* Rondani, 1873

15. *Heteropogon lehri* Richter, 1968

16. *Heteropogon mesasiaticus* Lehr, 1970

17. *Holopogon imbecillus* Loew, 1871

18. *Lamyra fimbriata* (Meigen, 1820)

19. *Lamyra fuliginosa* (Panzer, 1803)

20. *Laphria dizonias* Loew, 1847

21. *Lasiopogon avetianae* Richter, 1962

22. *Leptogaster armeniaca* Paramonov, 1930

23. *Leptogaster calceata* Engel, 1925

24. *Machimus erevanensis* Richter, 1963

25. *Molobratia egregia* (Loew, 1869)

26. *Neoitamus senectus* Richter, 1963

27. *Neomochtherus perplexus* (Becker, 1923)

28. *Neomochtherus rossicus* Engel, 1927

29. *Neopsilocurus hypopygialis* (Paramanov, 1930)

30. *Ommatius stackelbergi* (Richter, 1960)

31. *Pegesimallus mesasiaticus* (Lehr, 1958)

32. *Satanas gigas* Evermann, 1885

33. *Stenopogon callosus* (Pallas &Wiedemann, 1818)

34. *Stenopogon mollis* Loew, 1868

35. *Stenopogon nataliae* Richter, 1963

36. *Stichopogon callidus* Richter, 1966

37. *Stichopogon elegantulus* (Meigen & Wiedemann, 1820)

38. *Stichopogon venustus* Richter, 1963

39. *Tolmerus richterae* Lehr, 1981.

#### ﻿Bombyliidae

This is the largest and one of the best-researched Diptera families in Armenia. Many species have been described or recorded from Armenia by Paramonov (1940). The list below is based on the world catalogue published by Evenhuis and Greathead (1999; 2003; 2015).

1. *Amictus pictus* Loew, 1869

2. *Amictus validus* Loew, 1869

3. *Anastoecbus araxis* Paramonov, 1926

4. *Anastoechus caucasicus* Paramonov, 1930

5. *Anastoechus exalbidus* (Meigen & Wiedemann, 1820)

6. *Anastoechus fulvescens* Becker in Becker & Stein, 1913

7. *Anastoechus hyrcanus* (Pallas & Wiedemann, 1818)

8. *Anastoechus mylabricida* Zakhvatkin, 1934

9. *Anastoechus nigricirratus* Becker & Stein, 1913

10. *Anastoechus trisignatus* (Portschinsky, 1881)

11. *Anastoechus zimini* Paramonov, 1940

12. *Anthrax alagoezicus* Paramonov, 1935

13. *Anthrax anthrax* (Schrank, 1781)

14. *Anthrax armeniacus* Paramonov, 1935

15. *Anthrax chionostigma* Tsacas, 1962

16. *Anthrax erythrogaster* Paramonov, 1935

17. *Anthrax incitus* Paramonov, 1935

18. *Anthrax jazykovi* Zakhvaktin, 1934

19. *Anthrax leucogaster* Meigen & Wiedemann, 1820

20. *Anthrax monacha* (Sack, 1909)

21. *Anthrax pilosulus* Strobl, 1902

22. *Anthrax repetekianus* Paramonov, 1935

23. *Anthrax shelkovnikovi* Paramonov, 1935

24. *Anthrax sticticus* Klug, 1832

25. *Anthrax varius* Fabricius, 1794

26. *Anthrax virgo* Egger, 1859

27. *Antonia arenacea* Paramonov, 1933

28. *Antonia armeniaca* Paramonov, 1929

29. *Aphoebantus armeniacus* Paramonov, 1925

30. *Apolysis dolichorostris* Evenhuis & Greathead, 1999

31. *Beckerellus androgynus* (Loew, 1855)

32. *Bombomyia discoideus* (Fabricius, 1794)

33. *Bombomyia vertebralis* Dufour, 1833

34. *Bombylella atra* (Scopoli, 1763)

35. *Bombylisoma breviusculum* (Loew, 1855)

36. *Bombylisoma croaticum* (Kertész, 1901)

37. *Bombylisoma flavibarbum* (Loew, 1855)

38. *Bombylisoma melanocephalum* (Fabricius, 1794)

39. *Bombylisoma minimum* (Scopoli, 1771)

40. *Bombylisoma nigriceps* (Loew, 1862)

41. *Bombylisoma unicolor* (Loew, 1855)

42. *Bombylius alexanderi* Paramonov, 1940

43. *Bombylius ambustus* Pallas & Wiedemann, 1818

44. *Bombylius analis* Olivier, 1789

45. *Bombylius apicalis* Meigen, 1820

46. *Bombylius armeniacus* Paramonov, 1925

47. *Bombylius candidus* Loew, 1855

48. *Bombylius canescens* Mikan, 1796

49. *Bombylius cinerarius* Pallas & Wiedemann, 1818

50. *Bombylius cinerascens* Mikan, 1796

51. *Bombylius citrinus* Loew, 1855

52. *Bombylius discolor* Mikan, 1796

53. *Bombylius fimbriatus* Meigen, 1820

54. *Bombylius floccosus* Loew, 1857

55. *Bombylius fuscus* Fabricius, 1781

56. *Bombylius iranicus* Paramonov, 1940

57. *Bombylius major* Linnaeus, 1758

58. *Bombylius medius* Linnaeus, 1758

59. *Bombylius megacephalus* Portschinsky, 1887

60. *Bombylius minor* Linnaeus, 1758

61. *Bombylius modestus* Loew, 1873

62. *Bombylius mus* Bigot, 1862

63. *Bombylius niveus* Meigen, 1804

64. *Bombylius nubilus* Mikan, 1796

65. *Bombylius posticus* Fabricius, 1805

66. *Bombylius pumilus* Meigen, 1820

67. *Bombylius quadrifarius* Loew, 1855

68. *Bombylius repeteki* Paramonov, 1940

69. *Bombylius semifuscus* Meigen, 1820

70. *Bombylius shah* Paramonov, 1940

71. *Bombylius shelkovnikovi* Paramonov, 1926

72. *Bombylius testaceiventris* Paramonov, 1925

73. *Bombylius torquatus* Loew, 1855

74. *Bombylius trichurus* Wiedemann, 1818

75. *Bombylius venosus* Mikan, 1796

76. *Bombylius vlasovi* Paramonov, 1940

77. *Caecanthrax arabicus* (Macquart, 1840)

78. *Callostoma fascipenne* Macquart, 1840

79. *Chalcochiton pallasii* (Loew, 1856)

80. *Cononedys armenica* Paramonov, 1925

81. *Conophorus caucasicus* Zaitzev, 1960

82. *Conophorus glaucescens* (Loew, 1863)

83. *Conophorus nobilis* (Loew, 1873)

84. *Conophorus paraduncus* Paramonov, 1929

85. *Conophorus rjabovi* Paramonov, 1929

86. *Conophorus rossicus* Paramonov, 1929

87. *Conophorus syriacus* Paramonov, 1929

88. *Conophorus talyshensis* Zaitzev, 1960

89. *Conophorus virescens* (Fabricius, 1787)

90. *Cyllenia marginata* Loew, 1846

91. *Cyllenia rustica* (Rossi, 1790)

92. *Cytherea araxana* Paramonov, 1930

93. *Cytherea dispar* (Loew, 1873)

94. *Cytherea fenestrulata* (Loew, 1873)

95. *Cytherea elegans* Paramonov, 1930

96. *Cytherea infuscata* (Meigen, 1820)

97. *Cytherea melaleuca* (Loew, 1873)

98. *Cytherea obscura* (Fabricius, 1794)

99. *Cytherea persicanus* (Becker, 1903)

100. *Desmatoneura nivea* (Rossi, 1790)

101. *Eremyia transcaspicus* (Paramonov, 1924)

102. *Exhyalanthrax afer* (Fabricius, 1794)

103. *Exhyalanthrax intermedius* (Paramonov, 1927)

104. *Exhyalanthrax macrops* (Portschinsky, 1887)

105. *Exhyalanthrax melanchlaenus* (Loew, 1869)

106. *Exhyalanthrax muscarius* (Pallas & Wiedemann, 1818)

107. *Exhyalanthrax parvus* (Paramonov, 1927)

108. *Exhyalanthrax stigmula* (Klug, 1832)

109. *Exoprosopa capucina* (Fabricius, 1781)

110. *Exoprosopa cleomene* Egger, 1859

111. *Exoprosopa dedecor* Loew, 1871

112. *Exoprosopa dyonisii* Bigot, 1861

113. *Exoprosopa dispar* (Loew, 1869)

114. *Exoprosopa grandis* (Wiedemann in Meigen, 1820)

115. *Exoprosopa jacchus* (Fabricius, 1805)

116. *Exoprosopa lugens* Paramonov, 1928

117. *Exoprosopa minoides* Paramonov, 1928

118. *Exoprosopa minois* Loew, 1869

119. *Exoprosopa minos* (Meigen, 1804)

120. *Exoprosopa pectoralis* Loew, 1862

121. *Exoprosopa pleskei* Paramonov, 1928

122. *Exoprosopa rivularis* Meigen, 1820

123. *Exoprosopa rutila* Pallas & Wiedemann, 1818

124. *Geron auratus* Zaitzev, 1962

125. *Geron canescens* Zaitzev, 1962

126. *Geron griseus* Zaitzev, 1962

127. *Geron kozlovi* Zaitzev, 1972

128. *Geron krymensis* Paramonov, 1929

129. *Geron albifacies* Bezzi, 1924

130. *Hemipenthes caucasica* Zaitzev, 1966

131. *Hemipenthes exoprosopoides* Paramonov, 1928

132. *Hemipenthes hamifera* (Loew, 1854)

133. *Hemipenthes maura* (Linnaeus, 1758)

134. *Hemipenthes mischanensis* Paramonov, 1927

135. *Hemipenthes morio* (Linnaeus, 1758)

136. *Hemipenthes noscibilis* (Austen, 1937)

137. *Hemipenthes robusta* Zaitzev, 1966

138. *Hemipenthes subvelutina* Zaitzev, 1966

139. *Hemipenthes tushetica* Zaitzev, 1966

140. *Hemipenthes velutina* (Meigen, 1820)

141. *Heteralonia aeaca* (Meigen, 1804)

142. *Heteralonia aegina* (Wiedemann, 1828)

143. *Heteralonia araxana* (Paramonov, 1928)

144. *Heteralonia bagdadensis* (Macquart, 1840)

145. *Heteralonia kirgizorum* (Paramonov, 1928)

146. *Heteralonia latiuscula* (Loew, 1873)

147. *Heteralonia megerlei* (Meigen, 1820)

148. *Heteralonia melanoptera* (Pallas & Wiedemann, 1818)

149. *Heteralonia punctinervis* (Becker & Stein, 1913)

150. *Heteralonia subfasciata* (Engel, 1936)

151. *Heteralonia suffusa* (Klug, 1832)

152. *Legnotomyia erivanensis* (Paramonov, 1925)

153. *Legnotomyia singularis* (Macquart, 1846)

154. *Ligyra ferrea* (Walker, 1849)

155. *Lomatia armeniaca* Paramonov, 1924

156. *Lomatia belzebul* (Fabricius, 1794)

157. *Lomatia erinnys* Loew, 1869

158. *Lomatia flavitibia* Zaitzev, 1966

159. *Lomatia lachesis* Egger, 1859

160. *Lomatia lateralis* (Meigen, 1820)

161. *Lomatia meridiana* Paramonov, 1925

162. *Lomatia montana* Paramonov, 1924

163. *Lomatia persica* Paramonov, 1924

164. *Lomatia polyzona* Loew, 1869

165. *Lomatia rogenhoferi* Nowicki, 1868

166. *Lomatia shelkovnikovi* Paramonov, 1924

167. *Lomatia tisiphone* Loew in Schiner, 1860

168. *Mariobezzia armeniaca* Zaitzev, 1966

169. *Micomitra iris* (Loew, 1869)

170. *Micomitra shelkovnikovi* (Paramonov, 1928)

171. *Micomitra stupida* (Rossi, 1790)

172. *Oestranthrax karavajevi* Paramonov, 1931

173. *Oligodranes superbus* Engel, 1933

174. *Pachyanthrax telamon* (Loew, 1869)

175. *Petrorossia albula* Zaitzev, 1962

176. *Petrorossia caucasica* Zaitzev, 1962

177. *Petrorossia chraminensis* Zaitzev, 1962

178. *Petrorossia deserticola* Zaitzev, 1966

179. *Petrorossia flavipennis* Zaitzev, 1966

180. *Petrorossia fusca* Zaitzev, 1966

181. *Petrorossia hespera* (Rossi, 1790)

182. *Petrorossia lucidipennis* Zaitzev, 1966

183. *Petrorossia modesta* Zaitzev, 1966

184. *Petrorossia rufiventris* Zaitzev, 1966

185. *Phthiria canescens* Loew, 1846

186. *Phthiria grisea* Zaitzev, 1966

187. *Phthiria pulicaria* (Mikan, 1796)

188. *Phthiria quadrinotata* Loew, 1873

189. *Phthiria subnitens* Loew, 1846

190. *Phthiria umbripennis* Loew, 1846

191. *Phthiria vagans* Loew, 1846

192. *Prorachthes beckeri* Paramonov, 1927

193. *Prorachthes pleskei* Paramonov, 1927

194. *Satyramoeba hetrusca* (Fabricius, 1794)

195. *Spogostylum aethiops* (Fabricius, 1781)

196. *Spogostylum caucasicum* (Zaitzev, 1961)

197. *Spogostylum dubium* (Zaitzev, 1961)

198. *Spogostylum isis* (Meigen, 1820)

199. *Spogostylum rufulum* (Zaitzev, 1961)

200. *Spogostylum stackelbergi* (Paramonov, 1957)

201. *Spogostylum tripunctatum* (Wiedemann, 1820)

202. *Spogostylum zaitzevi* Evenhuis & Greathead, 1999

203. *Stomylomyia araxana* Paramonov, 1925

204. *Stomylomyia europaea* (Loew, 1869)

205. *Stomylomyia pusilla* Bezzi, 1925

206. *Systoechus autumnalis* (Pallas & Wiedemann, 1818)

207. *Systoechus ctenopterus* (Mikan, 1796)

208. *Systoechus gomezmenori* Andréu Rubio, 1959

209. *Systoechus gradatus* (Meigen & Wiedemann, 1820)

210. *Systoechus longirostris* Becker, 1916

211. *Systoechus lucidus* (Loew, 1855)

212. *Systoechus microcephalus* (Loew, 1855)

213. *Thyridanthrax dagniensis* Paramonov, 1933

214. *Thyridanthrax elegans* (Wiedemann, 1818)

215. *Thyridanthrax fenestratus* (Fallén, 1814)

216. *Thyridanthrax incanus* (Klug, 1832)

217. *Thyridanthrax lotus* Loew, 1869

218. *Thyridanthrax obliteratus* (Loew, 1862)

219. *Thyridanthrax perspicillaris* (Loew, 1869)

220. *Thyridanthrax polyphemus* (Wiedemann, 1820)

221. *Thyridanthrax rubriventris* (Paramonov, 1927)

222. *Toxophora epargyra* Hermann, 1907

223. *Toxophora fasciculata* (Villers, 1789)

224. *Toxophora psammophila* Paramonov, 1933

225. *Toxophora shelkovnikovi* Paramonov, 1933

226. *Triplasius pictus* (Panzer, 1794)

227. *Usia atrata* (Fabricius, 1798)

228. *Usia aurata* (Fabricius, 1794)

229. *Usia crinipes* Becker, 1906

230. *Usia transcaspica* Paramonov, 1950

231. *Veribubo albinus* (Becker & Stein, 1913)

232. *Veribubo desertus* (Zaitzev, 1966)

233. *Veribubo heteropterus* (Paramonov, 1927)

234. *Veribubo misellus* (Loew, 1869)

235. *Villa abbadon* (Fabricius, 1794)

236. *Villa brunnea* Becker, 1916

237. *Villa cana* Meigen, 1804

238. *Villa chorassani* (Becker & Stein, 1913)

239. *Villa cingulata* (Meigen, 1804)

240. *Villa claripennis* (Kowarz, 1868)

241. *Villa fasciata* (Meigen, 1804)

242. *Villa fasciculata* Becker, 1916

243. *Villa haesitans* Becker, 1916

244. *Villa halteralis* (Kowarz, 1883)

245. *Villa hottentota* (Linnaeus, 1758)

246. *Villa ixion* Fabricius, 1794

247. *Villa melanurus* (Loew, 1869)

248. *Villa modesta* (Meigen, 1820)

249. *Villa niphobleta* Loew, 1869

250. *Villa occulta* (Meigen & Wiedemann, 1820)

251. *Villa paniscus* Rossi, 1790

252. *Villa tomentosa* Becker, 1916

253. *Villa ventruosus* (Loew, 1869)

#### ﻿Calliphoridae

Several authors (Avetyan and Mirzoyan 1976; Rognes 2019; Cerretti et al. 2020; Gisondi et al. 2020) has dealt with the species of this family, which includes a number of species of considerable hygienic, medical, and veterinary importance.

1. *Bellardia bayeri* (Jacentkovský, 1937)

2. *Bellardia vulgaris* (Robineau-Desvoidy, 1830)

3. *Calliphora rohdendorfi* (Grunin, 1966)

4. *Calliphora vicina* Robineau-Desvoidy, 1830

5. *Calliphora vomitoria* (Linnaeus, 1758)

6. *Chrysomya albiceps* (Wiedemann, 1819)

7. *Lucilia ampullacea* Villeneuve, 1922

8. *Lucilia bufonivora* Moniez, 1876

9. *Lucilia caesar* (Linnaeus, 1758)

10. *Lucilia richardsi* Collin, 1926

11. *Lucilia sericata* (Meigen, 1826)

12. *Lucilia silvarum* (Meigen, 1826)

13. *Melinda gentilis* Robineau-Desvoidy, 1830

14. *Melinda viridicyanea* (Robineau-Desvoidy, 1830)

15. *Pollenia agneteae* Rognes, 2019

16. *Pollenia amentaria* (Scopoli, 1763)

17. *Pollenia bulgarica* Jacentkovský, 1939

18. *Pollenia dasypoda* Portschinsky, 1881

19. *Pollenia griseotomentosa* (Jacentkovský, 1944)

20. *Pollenia grunini* Rognes, 1988

21. *Pollenia mystica* Rognes, 1988

22. *Pollenia paragrunini* Rognes, 1988

23. *Pollenia pediculata* Macquart, 1834

24. *Pollenia rudis* (Fabricius, 1794)

25. *Pollenia viatica* Robineau-Desvoidy, 1830

26. *Protocalliphora azurea* (Fallén, 1817)

27. *Protophormia terraenovae* (Robineau-Desvoidy, 1830)

28. *Tromodesia shachrudi* (Rohdendorf, 1935)

#### ﻿Chamaemyiidae

The records from this family are from volume 9 of the Catalogue of Palearctic Diptera (Soós and Papp 1984).

1. *Chamaemyia emiliae* Tanasijtshuk, 1970

2. *Chamaemyia juncorum* (Fallén, 1823)

3. *Chamaemyia polystigma* (Meigen, 1830)

4. *Leucopis alticeps* Czerny, 1936

5. *Leucopis annulipes* Zetterstedt, 1848

6. *Leucopis compacta* Tanasijtshuk, 1972

7. *Leucopis glyphinivora* Tanasijtshuk, 1958

8. *Leucopis grandis* Tanasijtshuk, 1958

9. *Leucopis melanopus* Tanasijtshuk, 1959

10. *Leucopis ninae* Tanasijtshuk, 1966

11. *Leucopis palumbi* Rondani, 1872

12. *Leucopis pseudomelanopus* Tanasijtshuk, 1961

13. *Leucopis revisenda* Tanasijtshuk, 1970

14. *Leucopis rufitarsis* Tanasijtshuk, 1972

15. *Leucopis silesiaca* Egger, 1862

16. *Leucopis pallidolineata* Tanasijtshuk, 1961

17. *Parochthiphila coronata* (Loew, 1858)

18. *Parochthiphila nigripes* (Strobl, 1900)

19. *Parochthiphila phragmitis* Tanasijtshuk, 1963

#### ﻿Chloropidae

Several species of Chloropidae have been listed as agricultural pests in Armenia (Avetyan and Mirzoyan 1976) and a new species was described from Armenia by Dely-Draskovits (1979).

1. *Assuania thalhammeri* (Strobl, 1893)

2. *Chlorops pumilionis* (Bjerkander, 1778)

3. *Elachiptera cornuta* (Fallén, 1820)

4. *Lasiosina armeniaca* Dely-Draskovits, 1979

5. *Lasiosina cinctipes* (Meigen, 1830)

6. *Meromyza salitatrix* (Linnaeus, 1761)

7. *Oscinella frit* (Linnaeus, 1758)

8. *Oscinella pusilla* (Meigen, 1830)

#### ﻿Conopidae

Larvae of Conopidae are endoparasitoids of aculeate Hymenoptera and may be deleterious to honeybees (Soós and Papp 1984).

1. *Brachyceratias brevicornis* (Loew, 1847)

2. Conops (Conops) flavifrons Meigen, 1824

3. Conops (Conops) flavicauda (Bigot, 1880)

4. Conops (Conops) silacea Wiedemann, 1824

5. *Dalmannia aculeata* (Linnaeus, 1761)

6. *Dalmannia punctata* (Fabricius, 1794)

7. *Merziella longirostris* (Lyneborg, 1962)

8. *Myopa dorsalis* Fabricius, 1794

9. *Myopa morio* Meigen, 1804

10. *Myopa pallida* Kröber, 1916

11. *Myopotta pallipes* (Wiedemann, 1824)

12. *Physocephala abdominalis* (Kröber, 1915)

13. *Physocephala chrysorrhoea* (Meigen, 1824)

14. *Physocephala pusilla* (Meigen, 1804)

15. *Physocephala vittata* (Fabricius, 1794)

16. *Sicus ferrugineus* (Linnaeus, 1761)

17. *Thecophora distincta* (Wiedemann, 1824)

18. *Zodion cinereum* (Fabricius, 1794)

#### ﻿Curtonotidae

So far, only one record for this small family is known from Armenia (Roháček 2016).

1. *Curtonotum anus* (Meigen, 1830)

#### ﻿Dolichopodidae

Armenian records for this family have been listed in volume 7 of the Catalogue of Palearctic Diptera (Soós and Papp 1991).

1. *Campsicnemus curvipes* (Fallén, 1823)

2. *Campsicnemus umbripennis* Loew, 1856

3. *Chrysotus suavis* Loew, 1857

4. *Dolichopus armeniacus* Stackelberg, 1926

5. *Dolichopus excisus* Loew, 1859

6. *Dolichopus nubilus* Meigen, 1824

7. *Hercostomus apollo* (Loew, 1869)

8. *Hercostomus armenorum* Stackelberg, 1934

9. *Hercostomus caucasica* Stackelberg, 1934

10. *Hercostomus chaerophylli* (Meigen, 1824)

11. *Hercostomus chrysozygos* (Wiedemann, 1817)

12. *Hercostomus nigriplantis* (Stannius, 1831)

13. *Hercostomus phoebus* Parent, 1927

14. *Hercostomus rusticus* (Meigen, 1824)

15. *Hercostomus shelkovnikovi* Stackelberg, 1926

16. *Medetera armeniaca* Negrobov, 1972

17. *Medetera plumbella* Meigen, 1824

18. *Medetera verae* Negrobov, 1967

19. *Scellus paramonovi* Stackelberg, 1926

20. *Syntormon pumilus* (Meigen, 1824)

21. *Tachytrechus genualis* Loew, 1857

22. *Tachytrechus kowarzi* Mik, 1864

23. *Tachytrechus notatus* (Stannius, 1831)

24. *Tachytrechus petraeus* Loew, 1871

25. *Tachytrechus ripicola* Loew, 1857

#### ﻿Drosophilidae

Some species of Drosophilidae have been listed as agricultural pests in Armenia (Avetyan and Mirzoyan 1976).

1. *Drosophila funebris* (Fabricius, 1787)

2. *Drosophila melanogaster* Meigen, 1830

#### ﻿Empididae

The first records of Armenian Empididae are in volume 6 of the Catalogue of Palearctic Diptera (Soós and Papp 1989). Additional records and descriptions are by Shamshev and coauthors (Shamshev 2001; Shamshev 2016; Shamshev and Kustov 2018; Shamshev and Barták 2024).

1. *Dolichocephala monae* Joost, 1981

2. *Empis afipsiensis* Shamshev & Kustov, 2008

3. *Empis caucasica* Bezzi, 1909

4. *Empis crassa* Nowicki, 1868

5. *Empis sevanensis* Shamshev, 2002

6. *Empis tanasijtshuki* Shamshev & Kustov, 2018

7. *Empis tessellata* Fabricius, 1794

8. *Hilara sturmii* Wiedemann in Meigen 1822

9. *Rhamphomyia stepankubiki* Shamshev & Barták, 2024

10. *Rhamphomyia umbripennis* Meigen, 1822

#### ﻿Ephydridae

The earliest records for Armenia were given by Avetyan and Mirzoyan (1976). More recent records are from Mathis and Zatwarnicki (1995).

1. *Allotrichoma filiforme* Becker, 1896

2. *Diclasiopa lacteipennis* (Loew, 1862)

3. *Diclasiopa niveipennis* (Becker, 1896)

4. *Ditrichophora rampinii* (Canzoneri & Meneghini, 1975)

5. *Ephydra afghanica* Dahl, 1961

6. *Ephydra glauca* Meigen, 1830

7. *Hydrellia albiceps* (Meigen, 1824)

8. *Hydrellia griseola* Fallén, 1813

9. *Mosillus subsultans* (Fabricius, 1794)

10. *Notiphila dorsata* Stenhammar, 1844

11. *Notiphila graecula* Becker, 1926

12. *Notiphila nigricornis* Stenhammar, 1844

13. *Notiphila riparia* Meigen, 1830

14. *Notiphila venusta* Loew, 1856

15. *Parydra littoralis* (Meigen, 1830)

16. *Psilopa nitidula* Fallen, 1823

#### ﻿Fanniidae

The Fanniidae of Armenia have been enumerated in two faunistic papers by Pont (2015, 2017b).

1. *Fannia armata* (Meigen, 1826)

2. *Fannia barbata* (Stein, 1892)

3. *Fannia canicularis* (Linnaeus, 1761)

4. *Fannia caucasica* Pont, 2015

5. *Fannia cothurnata* (Loew, 1873)

6. *Fannia difficilis* (Stein, 1895)

7. *Fannia fuscula* (Fallén, 1825)

8. *Fannia genualis* (Stein, 1895)

9. *Fannia incisurata* (Zetterstedt, 1838)

10. *Fannia lepida* (Wiedemann, 1817)

11. *Fannia monilis* (Haliday, 1838)

12. *Fannia norvegica* Ringdahl, 1934

13. *Fannia ornata* (Meigen, 1826)

14. *Fannia parva* (Stein, 1895)

15. *Fannia polychaeta* (Stein, 1895)

16. *Fannia postica* (Stein, 1895)

17. *Fannia rondanii* (Strobl, 1893)

18. *Fannia scalaris* (Fabricius, 1794)

19. *Fannia serena* (Fallén, 1825)

20. *Fannia similis* (Stein, 1895)

#### ﻿Hybotidae

This family is represented by a very few species in Armenia (Soós and Papp 1989; Shamshev 2016).

1. *Crossopalpus aeneus* (Walker, 1871)

2. *Hybos vagans* Loew, 1874

3. *Platypalpus albiseta* (Panzer, 1806)

#### ﻿Lauxaniidae

This family is represented by only one species in Armenia (Soós and Papp 1984).

1. *Peplomyza intermedia* Remm, 1979

#### ﻿Micropezidae

This family is represented by only one species in Armenia (Soós and Papp 1984)

1. *Micropeza cingulata* Loew, 1868

#### ﻿Muscidae

The Muscidae of Armenia have been dealt with in a number of papers by Pont and colleagues, based on collections made by ACP in all parts of Armenia (Pont 2012, 2013a, 2022, 2023; Pont et al. 2005, 2011, 2012a, b). Records were summarised in Pont (2018). The new records listed below were found in the collections of the Zoological Institute of the Russian Academy of Sciences, St Petersburg, Russia, and in samples collected during the present project.

1. *Atherigona varia* (Meigen, 1826)

2. *Azelia aterrima* (Meigen, 1826)

3. *Azelia cilipes* (Haliday, 1838)

4. *Azelia monodactyla* Loew, 1874

5. *Azelia nebulosa* Robineau-Desvoidy, 1830

6. *Azelia triquetra* (Wiedemann, 1817)

7. *Azelia zetterstedtii* Rondani, 1866

8. *Coenosia agromyzina* (Fallén, 1825)

9. *Coenosia ambulans* Meigen, 1826

10. *Coenosia atra* Meigen, 1826

11. *Coenosia attenuata Stein in Becker, 1903

12. *Coenosia femoralis* (Robineau-Desvoidy, 1830)

13. *Coenosia gracilis* Stein, 1916

14. *Coenosia humilis* Meigen, 1826

15. *Coenosia infantula* Rondani, 1866

16. *Coenosia intermedia* (Fallén, 1825)

17. *Coenosia mollicula* (Fallén, 1825b)

18. *Coenosia nevadensis* Lyneborg, 1970

19. *Coenosia nigridigita* Rondani, 1866

20. *Coenosia octosignata* Rondani, 1866

21. *Coenosia oligochaeta Hennig, 1961

22. *Coenosia praetexta Pandellé, 1899

23. *Coenosia pulicaria* (Zetterstedt, 1845)

24. *Coenosia pumila* (Fallén, 1825)

25. *Coenosia pygmaea (Zetterstedt, 1845)

26. *Coenosia rufipalpis Meigen, 1826

27. *Coenosia testacea* (Robineau-Desvoidy, 1830)

28. *Coenosia tigrina* (Fabricius, 1775)

29. *Coenosia verralli* Collin, 1953

30. *Coenosia wernerae* Pont in Pont, Werner & Kachvoryan, 2005

31. *Dasyphora albofasciata* (Macquart, 1839)

32. *Dasyphora cyanella* (Meigen, 1826)

33. *Dasyphora cyanicolor* (Zetterstedt, 1845)

34. *Dasyphora meridionalis* Zimin, 1951

35. *Dasyphora penicillata* (Egger, 1865)

36. *Dasyphora pratorum* (Meigen, 1826)

37. *Dasyphora zimini* Hennig, 1963

38. *Drymeia caucasica* (Schnabl in Schnabl & Dziedzicki, 1911)

39. *Drymeia fasciculata* (Stein, 1916)

40. *Drymeia fratercula* Pont, 2022

41. *Drymeia setibasis* (Huckett, 1965)

42. *Drymeia sororcula* Pont, 2022

43. *Drymeia vicana* (Harris, 1780)

44. *Eginia ocypterata* (Meigen, 1826)

45. *Graphomya maculata* (Scopoli, 1763)

46. *Gymnodia polystigma* (Meigen, 1826)

47. *Haematobia irritans* (Linnaeus, 1758)

48. *Haematobia titillans* (Bezzi, 1907a)

49. *Haematobosca atripalpis* (Bezzi, 1895)

50. *Haematobosca stimulans* (Meigen, 1824)

51. *Hebecnema fumosa* (Meigen, 1826)

52. *Hebecnema nigra* (Robineau-Desvoidy, 1830)

53. *Hebecnema umbratica* (Meigen, 1826)

54. *Hebecnema vespertina* (Fallén, 1823)

55. *Helina arctata* Collin, 1953

56. *Helina atricolor* (Fallén, 1825)

57. *Helina balsaci* (Séguy, 1947)

58. *Helina celsa* (Harris, 1780)

59. *Helina chaetopyga* Malloch, 1921

60. *Helina cilipes* (Schnabl, 1902)

61. *Helina cinerella* (Wulp, 1867)

62. *Helina cothurnata* (Rondani, 1866)

63. *Helina decipiens* Mihályi, 1974

64. *Helina depuncta* (Fallén, 1825)

65. *Helina edita* Pont, 2012

66. *Helina evecta* (Harris, 1780)

67. *Helina fratercula (Zetterstedt, 1845)

68. *Helina impuncta* (Fallén, 1824)

69. *Helina irani* Zielke, 2017

70. *Helina karina* Pont, 2012

71. *Helina lasiophthalma* (Macquart, 1835)

72. *Helina latitarsis* Ringdahl, 1924

73. *Helina maculipennis* (Zetterstedt, 1845)

74. *Helina marisha* Pont, 2012

75. *Helina moedlingensis* (Schnabl in Schnabl & Dziedicki, 1911)

76. *Helina obscurata* (Meigen, 1826)

77. *Helina parcepilosa* (Stein, 1907)

78. *Helina pertusa (Meigen, 1826)

79. *Helina pubiseta* (Zetterstedt, 1845)

80. *Helina reversio* (Harris, 1780)

81. *Helina richardi* Pont, 2012

82. *Helina sexmaculata (Preyssler, 1791)

83. *Helina subvittata* (Séguy, 1923)

84. *Helina syracusana Hennig, 1957

85. *Helina tetrastigma* (Meigen, 1826)

86. *Helina trivittata* (Zetterstedt, 1860)

87. *Hydrotaea aenescens* (Wiedemann, 1830)

88. *Hydrotaea albipuncta* (Zetterstedt, 1845)

89. *Hydrotaea armipes* (Fallén, 1825)

90. *Hydrotaea borussica* Stein, 1899

91. *Hydrotaea capensis (Wiedemann, 1818)

92. *Hydrotaea cyrtoneurina* (Zetterstedt, 1845)

93. *Hydrotaea dentipes* (Fabricius, 1805)

94. *Hydrotaea floccosa* Macquart, 1835

95. *Hydrotaea glabricula* (Fallén, 1825)

96. *Hydrotaea himalayensis* Pont, 1975

97. *Hydrotaea hirticeps* (Fallén, 1824)

98. *Hydrotaea ignava* (Harris, 1780)

99. *Hydrotaea irritans* (Fallén, 1823)

100. *Hydrotaea meridionalis* Portschinsky, 1882

101. *Hydrotaea meteorica* (Linnaeus, 1758)

102. *Hydrotaea militaris (Meigen, 1826)

103. *Hydrotaea nidicola* Malloch, 1925

104. *Hydrotaea ringdahli* Stein, 1914

105. *Hydrotaea velutina* Robineau-Desvoidy, 1830

106. *Limnophora femoriseta* Pont, Vikhrev & Werner, 2011

107. *Limnophora latevittata* Schnabl in Schnabl & Dziedzicki, 1911

108. *Limnophora maculosa* (Meigen, 1826)

109. *Limnophora mediterranea* Pont in Pont et al. 2012

110. *Limnophora obsignata* (Rondani, 1866)

111. *Limnophora olympiae* Lyneborg, 1965

112. *Limnophora pandellei* Séguy, 1923

113. *Limnophora patellifera* (VilIeneuve, 1911)

114. *Limnophora pollinifrons* Stein, 1916

115. *Limnophora pulchriceps* (Loew, 1860)

116. *Limnophora riparia* (Fallén, 1824)

117. *Limnophora rufimana* (Strobl, 1893)

118. *Limnophora setinerva* Schnabl in Schnabl & Dziedzicki, 1911

119. *Limnophora tigrina* (Am Stein, 1860)

120. *Limnophora triangula* (Fallen, 1825)

121. *Lispe assimilis Wiedemann, 1824

122. *Lispe consanguinea* Loew, 1858

123. *Lispe longicollis* Meigen, 1826

124. *Lispe melaleuca* Loew, 1847

125. *Lispe nana* Macquart, 1835

126. *Lispe pygmaea* Fallén, 1825

127. *Lispe sericipalpis* Stein, 1904

128. *Lispe superciliosa Loew, 1861

129. *Lispe tentaculata* (De Geer, 1776)

130. *Lispocephala erythrocera* (Robineau-Desvoidy, 1830)

131. *Lispocephala spuria* (Zetterstedt, 1838)

132. *Lispocephala vitripennis Ringdahl, 1951

133. *Macrorchis meditata* (Fallén, 1825)

134. *Mesembrina meridiana* (Linnaeus, 1758)

135. *Mesembrina mystacea* (Linnaeus, 1758)

136. *Morellia aenescens Robineau-Desvoidy, 1830

137. *Morellia hortorum* (Fallén, 1817)

138. *Musca amita* Hennig, 1964

139. *Musca autumnalis* De Geer, 1776

140. *Musca domestica* Linnaeus, 1758

141. *Musca larvipara* Portschinsky, 1910

142. *Musca osiris* Wiedemann, 1830

143. *Musca tempestiva* Fallén, 1817

144. *Musca vitripennis* Meigen, 1826

145. *Muscina levida* (Harris, 1780)

146. *Muscina pascuorum* (Meigen, 1826)

147. *Muscina prolapsa* (Harris, 1780)

148. *Muscina stabulans* (Fallén, 1817)

149. *Mydaea ancilla* (Meigen, 1826)

150. *Mydaea corni (Scopoli, 1763)

151. *Mydaea detrita* (Zetterstedt, 1845)

152. *Mydaea humeralis* Robineau-Desvoidy, 1830

153. *Mydaea lateritia (Rondani, 1866)

154. *Mydaea nebulosa (Stein, 1893)

155. *Mydaea urbana* (Meigen, 1826)

156. *Myospila bimaculata* (Macquart, 1834)

157. *Myospila meditabunda* (Fabricius, 1781)

158. *Neomyia cornicina* (Fabricius, 1781)

159. *Neomyia viridescens* (Robineau-Desvoidy, 1830)

160. *Orchisia costata (Meigen, 1826)

161. *Phaonia adriani* Zinoviev, 1994

162. *Phaonia angelicae* (Scopoli, 1763)

163. *Phaonia cincta* (Zetterstedt, 1846)

164. *Phaonia errans* (Meigen, 1826)

165. *Phaonia gayaneae* Pont, 2018

166. *Phaonia gobertii* (Mik, 1881)

167. *Phaonia halterata* (Stein, 1893)

168. *Phaonia incana (Wiedemann, 1817)

169. *Phaonia kobica* Schnabl in Schnabl & Dziedzicki, 1911

170. *Phaonia modesta* Sorokina, 2015

171. *Phaonia pallida* (Fabricius, 1787)

172. *Phaonia palpata* (Stein, 1897)

173. *Phaonia perdita* (Meigen, 1830)

174. *Phaonia rufiventris (Scopoli, 1763)

175. *Phaonia serva* (Meigen, 1826)

176. *Phaonia signata* (Meigen, 1826)

177. *Phaonia subventa* (Harris, 1780)

178. *Phaonia trimaculata (Bouché, 1834)

179. *Phaonia tuguriorum* (Scopoli, 1763)

180. *Phaonia valida* (Harris, 1780)

181. *Polietes domitor* (Harris, 1780)

182. *Polietes lardarius* (Fabricius, 1781)

183. *Potamia littoralis* Robineau-Desvoidy, 1830

184. *Pyrellia secunda Zimin, 1951

185. *Pyrellia vivida* Robineau-Desvoidy, 1830

186. *Schoenomyza litorella* (Fallén, 1823)

187. *Spilogona carbonella* (Zetterstedt, 1845)

188. *Spilogona denigrata* (Meigen, 1826)

189. *Spilogona dispar* (Fallén, 1823)

190. *Spilogona meadei (Schnabl in Becker, 1915)

191. *Spilogona nora* Pont, 2013

192. *Spilogona paradispar* Pont, 2023

193. *Spilogona taeniata* (Stein, 1916)

194. *Stomoxys calcitrans* (Linnaeus, 1758)

195. *Thricops bukowskii* (Ringdahl, 1934)

196. *Thricops furcatus* (Stein, 1916)

197. *Thricops iliata* Pont, 2018

198. *Thricops longipes* (Zetterstedt, 1845)

199. *Thricops nigriabdominalis* Savage, 2003

200. *Thricops nigrifrons* (Robineau-Desvoidy, 1830)

201. *Thricops nigritellus* (Zetterstedt, 1838)

202. *Thricops rostratus* (Meade, 1882)

203. *Thricops semicinereus* (Wiedemann, 1817)

204. *Thricops simplex* (Wiedemann, 1817)

205. *Thricops sudeticus* (Schnabl, 1888)

206. *Thricops tomkovichi* Vikhrev in Vikhrev & Sorokina, 2009

207. *Thricops vaderi* Savage, 2003

208. *Ziminellia simplex* (Loew, 1857)

#### ﻿Mythicomyiidae

This family is another of the understudied families of Armenia. The records presented below are from the catalogue by Evenhuis (2002).

1. *Cyrtisiopsis melleus* (Loew, 1856)

2. *Platypygus bellus* Loew, 1869

3. *Platypygus kurdorum* Paramonov, 1929

4. *Platypygus melinoproctus* Loew, 1873

5. *Platypygus ridibundus* (Costa, 1863)

#### ﻿Opomyzidae

This family is represented by two species in Armenia, which are agricultural pests (Avetyan and Mirzoyan 1976).

1. *Geomyza tripunctata* (Fallen, 1823)

2. *Opomyza florum* (Fabricius, 1794)

#### ﻿Phaeomyiidae

This small family, recently separated from the Sciomyzidae, contains species that are parasitoids of millipedes (Diplopoda) (Soós and Papp 1984).

1. *Pelidnoptera nigripennis* (Fabricius, 1794)

#### ﻿Piophilidae

The only Armenian record for this family has been found in the list of pest species by Avetyan and Mirzoyan (1976).

1. *Piophila casei* (Linnaeus, 1758)

#### ﻿Platystomatidae

The records from this family were found in volume 9 of the Catalogue of Palearctic Diptera (Soós and Papp 1984). The last species was from Avetyan and Mirzoyan (1976) where the family was ﻿Platystotidae.

1. *Platystoma bipilosum* Portschinsky, 1875

2. *Platystoma chrysotoxum* Hendel, 1913

3. *Platystoma malatiense* Hennig, 1945

4. *Platystoma punctiventre* Portschinsky, 1875

5. Platystoma seminationis ssp. rufimana Loew, 1873

6. *Platystomata gilvipes* Loew, 1868

7. *Platystoma rufipes* Meigen, 1826

#### ﻿Rhinophoridae

Rhinophorid larvae are endoparasitoids of woodlice. One species has been recorded from Armenia (Cerretti et al. 2020).

1. *Tromodesia shachrudi* (Rohdendorf, 1935)

#### ﻿Sarcophagidae

Many records of Sarcophagidae from Armenia date from Rohdendorf (1937) and, more recently, from a number of papers by Yu.G. Verves. These have been summarised and supplemented by the world catalogue by Pape (1996).

1. *Amobia pelopei* Rondani, 1859

2. *Angiometopa falleni* Pape, 1986

3. *Apodacra ljudmilae* Verves, 1980

4. *Apodacra rohdendorfi* Verves, 1980

5. *Blaesoxipha grisea* (Meigen, 1926)

6. *Blaesoxipha richterae* Rohdendorf & Verves, 1977

7. *Blaesoxipha shelkovnikovi* Rohdendorf, 1937

8. *Brachicoma devia* (Fallén, 1820)

9. *Craticulina tabaniformis* (Fabricius, 1805)

10. *Metopia argyrocephala* (Meigen, 1824)

11. *Metopia campestris* (Fallén, 1810)

12. *Miltogramma chivae* Rohdendorf, 1935

13. *Miltogramma occipitalis* Pandellé, 1895

14. *Miltogramma oestracea* (Fallén, 1820)

15. *Miltogramma punctata* Meigen, 1824

16. *Miltogramma ruficornis* Meigen, 1824

17. *Miltogramma rutilans* Meigen, 1824

18. *Miltogramma taeniata* Meigen, 1824

19. *Miltogramma testaceifrons* (Roser, 1840)

20. *Nyctia halterata* (Panzer, 1798)

21. *Paramacronychia flavipalpis* (Girschner, 1881)

22. *Phylloteles pictipennis* Loew, 1844

23. *Protomiltogramma fasciata* (Meigen, 1824)

24. *Protomiltogramma jaxartiana* (Rohdendorf, 1927)

25. *Pterella grisea* (Meigen, 1824)

26. *Pterella melanura* (Meigen, 1824)

27. *Pterella penicillaris* (Rondani, 1865)

28. *Ravinia pernix* (Harris, 1780)

29. *Sarcophaga aegyptica* Salem, 1935

30. *Sarcophaga africa* Wiedemann, 1824

31. *Sarcophaga albiceps* Meigen, 1826

32. *Sarcophaga ancilla* Rondani, 1865

33. *Sarcophaga aratrix* Pandellé, 1896

34. *Sarcophaga argyrostoma* (Robineau-Desvoidy, 1830)

35. *Sarcophaga armenica* (Rohdendorf, 1937)

36. *Sarcophaga bulgarica* (Enderlein, 1936)

37. *Sarcophaga caerulescens* Zetterstedt, 18938

38. *Sarcophaga carnaria* (Linnaeus, 1758)

39. *Sarcophaga crassipalpis* Macquart, 1839

40. *Sarcophaga cucullans* Pandellé, 1896

41. *Sarcophaga dreyfusi* (Lehrer, 1994)

42. *Sarcophaga haemorrhoides* (Böttcher, 1913)

43. *Sarcophaga incisilobata* Pandellé, 1896

44. *Sarcophaga jacobsoni* (Rohdendorf, 1937)

45. *Sarcophaga lehmanni* Muller, 1922

46. *Sarcophaga lunigera* (Böttcher, 1914)

47. *Sarcophaga lypai* (Verves, 1977)

48. *Sarcophaga maculata* Meigen, 1835

49. *Sarcophaga mehadiensis* Böttcher, 1912

50. *Sarcophaga melanura* Meigen, 1826

51. *Sarcophaga mutila* Villeneuve, 1912

52. *Sarcophaga nigriventris* Meigen, 1826

53. *Sarcophaga olsoufjevi* (Rohdendorf, 1937)

54. *Sarcophaga pachyura* (Rohdendorf, 1937)

55. *Sarcophaga paramonovi* (Rohdendorf, 1937)

56. *Sarcophaga pontica* (Rohdendorf, 1937)

57. *Sarcophaga portschinskyi* (Rohdendorf, 1937)

58. *Sarcophaga protuberans* Pandellé, 1896

59. *Sarcophaga recta* (Rohdendorf, 1937)

60. *Sarcophaga setinervis* Rondani, 1860

61. *Sarcophaga sexpunctata* (Fabricius, 1805)

62. *Sarcophaga turana* (Rohdendorf, 1937)

63. *Sarcophaga uliginosa* Kramer, 1908

64. *Sarcophila meridionalis* Verves, 1982

65. *Senotainia albifrons* (Rondani, 1859)

66. *Senotainia armenica* Rohdendorf, 1935

67. *Senotainia conica* (Fallén, 1810)

68. *Senotainia tricuspis* (Meigen, 1824)

69. *Sphecapatoclea armenica* (Rohdendorf, 1975)

70. *Sphecapatodes kaszabi* Rohdendorf & Verves, 1980

71. *Sphenometopa bifasciata* (Brauer & Bergenstamm, 1891)

72. *Sphenometopa claripennis* (Villeneuve, 1933)

73. *Sphenometopa fastuosa* (Meigen, 1824)

74. *Sphenometopa mannii* (Brauer & Bergenstamm, 1891)

75. *Taxigramma armeniaca* (Verves, 1980)

76. *Taxigramma elegantula* (Zetterstedt, 1844)

77. *Taxigramma heteroneura* (Meigen, 1830)

78. *Taxigramma multipunctata* (Rondani, 1859)

79. *Taxigramma stictica* (Meigen, 1830)

80. *Wohlfahrtia balassogloi* (Portschinsky, 1881)

81. *Wohlfahrtia bella* (Macquart, 1839)

82. *Wohlfahrtia magnifica* (Schiner, 1862)

83. *Wohlfahrtia nuba* (Wiedemann, 1830)

#### ﻿Scathophagidae

There are only two published records for this family, by Ozerov and Krivosheina (2020). The new records from Armenia are based on collections made by ACP in all parts of Armenia and deposited in the Natural History Museum, London (UK).

1. *Cordilura albilabris* (Fabricius, 1805)

2. *Cordilura albipes* Fallén, 1819

3. *Cordilura pubera (Linnaeus, 1758)

4. *Norellisoma lesgiae (Becker, 1894)

5. *Norellisoma spinimanum (Fallén, 1819)

6. *Scathophaga inquinata Meigen, 1826

7. *Scathophaga stercoraria (Linnaeus, 1758)

8. *Spaziphora hydromyzina (Fallén, 1819)

#### ﻿Sciomyzidae

The historical records for the family are from volume 9 of the Catalogue of Palearctic Diptera (Soós and Papp 1984). The new records from Armenia are based on collections made by ACP in all parts of Armenia and deposited in the Natural History Museum, London (UK).

1. *Antichaeta analis* (Meigen, 1830)

2. *Coremacera catenata* (Loew, 1847)

3. Coremacera marginata ssp. pontica (Elberg, 1968)

4. *Dichaetophora obliterata (Fabricius, 1805)

5. *Hydromya dorsalis (Fabricius, 1775)

6. *Knutsonia albiseta* (Scopoli, 1763)

7. *Knutsonia turcestanica* (Hendel, 1903)

8. *Limnia unguicornis* (Scopoli, 1763)

9. *Pherbellia argyra Verbeke, 1967

10. *Pherbellia cinerella* (Fallén, 1820)

11. *Sepedon sphegea* (Fabricius, 1775)

12. *Sepedon spinipes* (Scopoli, 1763)

13. *Tetanocera arrogans Meigen, 1830

14. *Tetanocera elata (Fabricius, 1781)

15. *Tetanocera montana* Day, 1881

16. *Trypetoptera punctulata* (Scopoli, 1763)

#### ﻿Sepsidae

The Sepsidae of Armenia have been enumerated in two faunistic papers by Pont (2013b, 2017a).

1. *Nemopoda nitidula* (Fallén, 1820)

2. *Saltella nigripes* Robineau-Desvoidy, 1830

3. *Saltella sphondylii* (Schrank, 1803)

4. *Sepsis barbata* Becker, 1907

5. *Sepsis biflexuosa* Strobl, 1893

6. *Sepsis cynipsea* (Linnaeus, 1758)

7. *Sepsis flavimana* Meigen, 1826

8. *Sepsis fulgens* Meigen, 1826

9. *Sepsis luteipes* Melander & Spuler, 1917

10. *Sepsis neocynipsea* Melander & Spuler, 1917

11. *Sepsis orthocnemis* Frey, 1908

12. *Sepsis punctum* (Fabricius, 1794)

13. *Sepsis thoracica* (Robineau-Desvoidy, 1830)

14. *Sepsis violacea* Meigen, 1826

15. *Susanomira caucasica* Pont, 1987

16. *Themira annulipes* (Meigen, 1826)

17. *Themira lucida* (Staeger, 1844)

18. *Themira minor* (Haliday, 1833)

#### ﻿Sphaeroceridae

The records for this family are from Roháček (2001).

1. *Ceroptera rufitarsis* (Meigen, 1830)

2. *Leptocera nigra* Olivier, 1813

3. *Opacifrons coxata* (Stenhammar, 1855)

4. *Pseudocollinella humida* (Haliday, 1836)

5. *Rachispoda gel* (Papp, 1978)

6. *Rachispoda modesta* (Duda, 1924)

#### ﻿Stratiomyidae

The records for this family are from the world catalogue by Woodley (2001)

1. *Actina chalybea* Meigen, 1804

2. *Adoxomyia portschinskii* (Pleske, 1903)

3. *Beris kovalevi* Rozkošný & Nartshuk, 1980

4. *Chloromyia speciosa* (Macquart, 1834)

5. *Hermionella seguyi* Pleske, 1925

6. *Lasiopa caucasica* (Pleske, 1901)

7. *Nemotelus argentifer* Loew, 1846

8. *Nemotelus pantherinus* (Linnaeus, 1758)

9. *Nemotelus rufiventris* Portschinsky, 1887

10. *Odontomyia hydroleon* (Linnaeus, 1758)

11. *Oxycera analis* Meigen & Wiedemann, 1822

12. *Oxycera pardalina* Meigen, 1822

13. *Pycnomalla splendens* (Fabricius, 1787)

14. *Sargus cuprarius* (Linnaeus, 1758)

15. *Stratiomys armeniaca* Bigot, 1879

16. *Stratiomys cenisia* Meigen, 1822

17. *Stratiomys chamaeleon* (Linnaeus, 1758)

18. *Stratiomys longicornis* (Scopoli, 1763)

19. *Stratiomys nobilis* Loew, 1871

20. *Stratiomys ruficornis* Macquart, 1838

21. *Stratiomys singularia* (Harris, 1778)

22. *Stratiomys viridis* Paramonov, 1926

#### ﻿Tabanidae

Most of the records for this family are from northern regions of Armenia (Hovhannisyan et al. 2023). Earlier records from Armenia are from faunistic work of Olsufiev (1937).

1. *Atylotus fulvus* (Meigen, 1804)

2. Chrysops flavipes ssp. abdominalis Kröber, 1920

3. Chrysops flavipes ssp. flavipes Meigen, 1804

4. *Chrysops caecutiens* (Linnaeus, 1758)

5. *Chrysops sejunctus* Szilady, 1919

6. *Haematopota ocelligera* (Kröber, 1922)

7. *Haematopota subcylindrica* Pandellé, 1883

8. *Hybomitra caucasica* (Enderlein, 1925)

9. *Hybomitra muehlfeldi* (Brauer, 1880)

10. *Hybomitra popovi* (Olsufjev, 1937)

11. *Philipomyia aprica* (Meigen, 1820)

12. *Silvius caucasicus* Olsufjev, 1937

13. *Tabanus armeniacus* (Kröber, 1928)

14. *Tabanus atropathenicus* Olsufjev, 1937

15. Tabanus bifarius ssp. bifarius Loew, 1858

16. Tabanus bromius ssp. bromius Linnaeus, 1758

17. Tabanus bromius ssp. flavofemoratus Strobl, 1909

18. *Tabanus capito* Olsufjev, 1937

19. *Tabanus cordiger* Meigen & Wiedemann,1820

20. *Tabanus glaucopis* Meigen, 1820

21. *Tabanus hauseri* Olsufjev, 1967

22. Tabanus indrae ssp. indrae Hauser, 1939

23. Tabanus indrae ssp. vappa Bogatchev & Samedov, 1949

24. *Tabanus miki* Brauer,1880

25. *Tabanus olsufjevi* Hauser, 1960

26. *Tabanus portschinskii* Olsufjev, 1937

27. *Tabanus prometheus* Szilady, 1923

28. Tabanus quatuornotatus ssp. quatuornotatus Meigen, 1820

29. *Tabanus rupium* (Brauer, 1880)

30. *Tabanus semiargenteus* Olsufjev, 1937

31. *Tabanus shelkovnikovi* Paramonov,1933

32. *Tabanus spectabilis* Loew, 1858

33. *Tabanus tergestinus* Egger, 1859

34. *Therioplectes carabaghensis* Portschinsky, 1877

35. Therioplectes tricolor ssp. tricolor Zeller, 1842

36. Tabanus (Ochrops) fulvus Meigen, 1804

#### ﻿Tachinidae

All the records for this family are from O’Hara et al. (2020).

1. *Admontia maculisquama* (Zetterstedt, 1859)

2. *Baumhaueria goniaeformis* (Meigen, 1824)

3. *Bessa parallela* (Meigen, 1824)

4. *Billaea biserialis* (Portschinsky, 1881)

5. *Billaea intermedia* (Portschinsky, 1881)

6. *Billaea trochanterata* Mesnil, 1970

7. *Bithia achanthophora* (Rondani, 1861)

8. *Blepharomyia pagana* (Meigen, 1824)

9. *Brachychaeta petiolata* Mesnil, 1953

10. *Brachicheta strigata* (Meigen, 1824)

11. *Cadurcia casta* (Rondani, 1861)

12. *Carcelia gnava* (Meigen, 1824)

13. *Cistogaster mesnili* (Zimin, 1966)

14. *Clemelis pullata* (Meigen, 1824)

15. *Compsilura concinnata* (Meigen, 1824)

16. *Cylindromyia brevicornis* (Loew, 1844)

17. *Cylindromyia pusilla* Meigen, 1824)

18. *Datvia deserticola* Richter, 1972

19. Dinera fuscata ssp. occidentalis Ziegler in Ziegler, Lutovinovas & Zhang, 2016

20. *Drino inconspicua* (Meigen, 1830)

21. *Erynniopsis antennata* (Rondani, 1861)

22. *Exorista larvarum* (Linnaeus, 1758)

23. *Exorista rossica* Mesnil, 1960

24. *Exorista segregata* (Rondani, 1859)

25. *Germaria sesiophaga* Richter, 1987

26. *Gonia asiatica* (Rohdendorf, 1928)

27. *Gymnosoma desertorum* (Rohdendorf, 1947)

28. *Hypovoria hilaris* Villeneuve, 1913

29. *Leskia erevanica* Richter, 1974

30. *Leucostoma anthracina* (Meigen, 1824)

31. *Linnaemya comta* (Fallén, 1810)

32. *Linnaemya olsufjevi* Zimin, 1954

33. *Lomachantha rufitarsis* Villeneuve, 1912

34. *Lydella thompsoni* Herting, 1959

35. *Lydina aenea* (Meigen, 1824)

36. *Masicera sphingivora* (Robineau-Desvoidy, 1830)

37. *Macquartia tessellum* (Meigen, 1824)

38. *Minthodes atra* (Kugler, 1971)

39. *Minthodes picta* (Zetterstedt, 1844)

40. *Minthodes pictipennis* Brauer & Bergenstamm, 1889

41. *Naira nata* Richter, 1970

42. *Nanoplagia hilfii* (Strobl, 1902)

43. *Nemorilla floralis* (Fallén, 1810)

44. *Neoemdenia mirabilis* Mesnil, 1953

45. *Ocytata pallipes* (Fallén, 1820)

46. *Pales pavida* (Meigen, 1824)

47. *Panzeria armeniaca* (Richter, 1972)

48. *Parasetigena silvestris* (Robineau-Desvoidy, 1863)

49. *Paratryphera mesnili* Herting, 1977

50. *Platymya antennata* (Brauer & Bergenstamm, 1891)

51. *Peletieria paramonovi* Zimin, 1935

52. *Periscepsia plorans* (Rondani, 1861)

53. *Peteina erinaceus* (Fabricius, 1794)

54. *Phorocera grandis* (Rondani, 1859)

55. *Rossimyiops longicornis* (Kugler, 1972)

56. *Smidtia latifrons* (Richter, 1972)

57. *Sonaca moderata* (Herting, 1979)

58. *Sturmia bella* (Meigen, 1824)

59. *Tachina fera* (Linnaeus, 1761)

60. *Tachina praeceps* Meigen, 1824

61. *Tachina rohdendorfi* Zimin, 1935

62. *Thelymorpha marmorata* (Fabricius, 1805)

63. *Townsendiellomyia nidicola* (Townsend, 1908)

64. *Triarthria legeri* (Villeneuve, 1908)

65. *Zenillia libatrix* (Panzer, 1797)

66. *Zeuxia erythraea* (Egger, 1856)

67. *Zeuxia roederi* (Villeneuve, 1932)

68. *Zeuxia tricolor* (Portschinsky, 1881)

#### ﻿Tephritidae

Most of the species of this family were listed by Avetyan and Mirzoyan (1976), with a few recent additions by Mohamadzade and Nozari (2010), and more recent records have been given by Evstigneev and Glukhova (2022).

1. *Acanthiophilus helianthi* (Rossi, 1794)

2. *Carpomyia schineri* Loew, 1856

3. *Goniurellia tridens* (Hendel, 1910)

4. *Hypenidium roborowskii* (Becker, 1907)

5. *Myiopardalis pardalina* (Bigot, 1891)

6. *Rhagoletis berberidis* Jermy, 1961

7. *Rhagoletis cerasi* (Linnaeus, 1758)

8. *Rhagoletis meigenii* Loew, 1844

9. *Trupanea amoena* (Frauenfeld, 1857)

10. *Trupanea stellata* (Fuesslin, 1775)

11. *Urophora terebrans* (Loew, 1850)

12. *Xyphosia laticauda* (Meigen, 1826)

#### ﻿Therevidae

All the records for the family are from volume 6 of the Catalogue of Palearctic Diptera (Soós and Papp 1989).

1. *Actorthia lacteipennis* (Becker, 1912)

2. *Ammothereva splendida* (Kröber, 1912)

3. *Haplosathe richterae* Lyneborg & Zaitzev, 1980

4. *Thereva affinis* Kröber, 1913

5. *Thereva brunninervis* Kröber, 1913

6. *Thereva caucasica* Kröber, 1913

7. *Thereva ordubadica* Paramonov, 1927

8. *Thereva panotshinii* Paramonov, 1927

#### ﻿Ulidiidae

The species listed here are from volume 9 of the Catalogue of Palearctic Diptera (Soós and Papp 1984) where the family name was given as Otitidae.

1. *Dorycera caucasica* Hendel, 1910

2. *Dorycera maculipennis* Macquart, 1844

### ﻿Nematocera

The suborder “Nematocera” is represented by 17 families in Armenia.

#### ﻿Anisopodidae

Only 2 species have been recorded from Armenia, by Oboňa et al. (2017).

1. *Sylvicola cinctus* (Fabricius, 1787)

2. *Sylvicola stackelbergi* Krivosheina & Menzel, 1998

#### ﻿Bibionidae

The first record for this family was by Avetyan and Mirzoyan (1976), and recent additions have been by Oboňa et al. (2017).

1. *Bibio consanguineus* Loew, 1869

2. *Bibio hortulanus* (Linnaeus, 1758)

3. *Dilophus bispinosus* Lundström, 1913

4. *Dilophus febrilis* (Linnaeus, 1758)

#### ﻿Blephariceridae

There is only a single record of this family in Armenia (Zwick 1988).

1. *Liponeura tarnogradskyi* Bischoff, 1930

#### ﻿Cecidomyiidae

This family of gall midges is one of the better-studied families of Diptera in Armenia (Mirumyan 2011; Mirumian and Skuhravá 2017, 2022; Gagné and Jaschhof 2021; Babayan 2022; Fedotova et al. 2022; Hovhannisyan et al. 2023).

1. *Ametrodiplosis crassinerva* (Kieffer, 1901)

2. *Apiomyia bergenstammi* (Wachtl, 1882)

3. *Aschistonyx carpinicolus* Rubsaamen, 1917

4. *Asphondylia hornigi* Wachtl, 1881

5. *Asphondylia pruniperda* Rondani, 1867

6. *Asphondylia scrophulariae* (Schiner, 1856)

7. *Asphondylia serpylli* Kieffer, 1898

8. *Asphondylia verbasci* (Vallot, 1827)

9. *Baldratia terteriani* (Mamaev & Mirumian, 1990)

10. *Bayeriola thymicola* (Kieffer, 1888)

11. *Blastomyia origani* (Tavares, 1901)

12. *Bremiola onobrychidis* (Bremi, 1847)

13. *Careopalpis harenosa* (Möhn, 1971)

14. *Careopalpis suaedae* (Fedotova, 1983)

15. *Careopalpis suaedicola* Fedotova, 1985

16. *Cephalaromyia capituli* Skuhravá, 2017 [invalid]

17. *Clinodiplosis cilicrus* (Kieffer, 1889)

18. *Contarinia baeri* (Prell, 1931)

19. *Contarinia campanulae* (Kieffer, 1895)

20. *Contarinia craccae* Kieffer, 1897

21. *Contarinia desertorum* Marikovskii, 1961

22. *Contarinia heraclei* (Rübsaamen, 1889)

23. *Contarinia inulicola* Stelter, 1965

24. *Contarinia lepidii* Kieffer, 1909

25. *Contarinia medicaginis* Kieffer, 1895

26. *Contarinia molluginis* (Rübsaamen, 1889)

27. *Contarinia onobrychidis* Kieffer, 1895

28. *Contarinia pyrivora* (Riley, 1886)

29. *Contarinia rhamni* (Rübsaamen, 1892)

30. *Contarinia steini* (Karsch, 1881)

31. *Contarinia tritici* (Kirby, 1798)

32. *Contarinia zygophylliflorae* Fedotova, 1990

33. *Cystiphora taraxaci* (Kieffer, 1888)

34. *Dasineura acrophila* (Winnertz, 1853)

35. *Dasineura aparines* (Kieffer, 1889)

36. *Dasineura asperulae* (Löw, 1875)

37. *Dasineura auritae* (Rübsaamen, 1915)

38. *Dasineura bayeri* (Rubsaamen, 1914)

39. *Dasineura clematidina* (Kieffer, 1913)

40. *Dasineura engstfeldi* (Rübsaamen, 1889)

41. *Dasineura euphorbiarum* (Kieffer, 1909)

42. *Dasineura fairmairei* (Kieffer, 1897)

43. *Dasineura foliumcrispans* (Rubsaamen, 1895)

44. *Dasineura fraxinea* Kieffer, 1907

45. *Dasineura fraxini* (Bremi, 1847)

46. *Dasineura gentneri* (Pritchard, 1953)

47. *Dasineura gleditchiae* (Osten Sacken, 1866)

48. *Dasineura hyperici* (Bremi, 1847)

49. *Dasineura irregularis* (Bremi, 1847)

50. *Dasineura lathyri* (Kieffer, 1909)

51. *Dasineura leguminicola* (Linther, 1879)

52. *Dasineura mali* (Kieffer, 1894)

53. *Dasineura papaveris* (Winnertz, 1853)

54. *Dasineura pyri* (Bouché, 1847)

55. *Dasineura rosae* (Bremi, 1847)

56. *Dasineura rubella* (Kieffer, 1896)

57. *Dasineura spadicea* (Rübsaamen, 1917)

58. *Dasineura tortrix* (Löw, 1877)

59. *Dasineura traili* (Kieffer, 1909)

60. *Dasineura trifolii* (Löw, 1874)

61. *Dasineura urticae* (Perris, 1840)

62. *Dasineura viciae* (Kieffer, 1888)

63. *Dasyneuriola salicorniae* (Fedotova, 1984)

64. *Dasyneuriola suaedae* Marikovskij, 1961

65. *Euphorbomyia loewi* (Mik, 1882)

66. *Geocrypta braueri* (Handlirsch, 1884)

67. *Geocrypta heterophylli* (Rübsaamen, 1914)

68. *Halodiplosis araratica* Mirumian, 1991

69. *Hartigiola annulipes* (Hartig, 1839)

70. *Iteomyia capreae* (Winnertz, 1853)

71. *Jaapiella bryoniae* (Bouché, 1847)

72. *Jaapiella cirsiicola* Rübsaamen, 1915

73. *Jaapiella loticola* (Rübsaamen, 1889)

74. *Jaapiella medicaginis* (Rübsaamen, 1912)

75. *Jaapiella parvula* (Liebel, 1889)

76. *Jaapiella rubiae* Fedotova, 1987

77. *Jaapiella thalictri* (Rübsaamen, 1895)

78. *Jaapiella volvens* Rübsaamen, 1917

79. *Janetiella convolvuli* Mirumian &Skuhravá, 2017

80. *Janetiella thymi* (Kieffer, 1888)

81. *Kochiomyia kochiae* (Kieffer, 1909)

82. *Macrodiplosis pustularis* (Bremi, 1847)

83. *Macrodiplosis roboris* (Hardy, 1854)

84. *Macrolabis heraclei* (Kaltenbach, 1862)

85. *Mayetiola destructor* (Say, 1817)

86. *Mikiola fagi* (Hartig, 1839)

87. *Mikiola orientalis* Kieffer, 1909

88. *Neolasioptera odontonemae* Möhn, 1964

89. *Obolodiplosis robiniae* (Haldeman, 1847)

90. *Ozirhincus millefolii* (Wachtl, 1884)

91. *Phegomyia fagicola* (Kieffer, 1901)

92. *Polygonomyia atraphaxiflorae* (Fedotova, 1984)

93. *Primofavilla initialis* Mamaev, 1972

94. *Psectrosema barbatum* (Marikovskiy, 1961)

95. *Psectrosema dentipes* (Marikovskiy, 1961)

96. *Pseudokochiomyia camphorosmae* (Fedotova, 1984)

97. *Pseudokochiomyia mesasiatica* (Fedotova, 1982)

98. *Pseudokochiomyia panderiae* Fedotova, 1992

99. *Pseudokochiomyia vicina* (Marikovskiy, 1961)

100. *Rabdophaga gemmicolata* Gagné, 2004

101. *Rabdophaga heterobia* (Loew, 1850)

102. *Rabdophaga iteobia* (Kieffer, 1890)

103. *Rabdophaga marginemtorquens* (Bremi, 1847)

104. *Rabdophaga rosaria* (Loew, 1850)

105. *Rabdophaga saliciperda* (Dufour, 1841)

106. *Rabdophaga salicis* (Schrank, 1803)

107. *Rabdophaga terminalis* (Loew, 1850)

108. *Rabdophaga salicis* (Schrank, 1803)

109. *Rhopalomyia artemisiae* (Bouché, 1834)

110. *Rhopalomyia baccarum* (Wachtl, 1883)

111. *Rhopalomyia campestris* (Rübsaamen, 1915)

112. *Rhopalomyia heteropalpis* (Marikovskiy in Marikovskiy & Moiseeva, 1964)

113. *Rhopalomyia hispanica* Tavares, 1904

114. *Rhopalomyia millefolii* (Loew, 1850)

115. *Rhopalomyia navasi* (Tavares, 1904)

116. *Rhopalomyia syngenesiae* (Loew, 1850)

117. *Rhopalomyia tubifex* (Bouché, 1847)

118. *Sophoromyia armenica* Mamaev & Mirumian, 1989

119. *Spurgia euphorbiae* (Vallot, 1827)

120. *Stefaniella hilversidae* Mamaev, 1972

121. *Stefaniola excelsa* Möhn, 1971

122. *Tavolgomyia karelini* (Fedotova, 1982)

123. *Wachtliella persicariae* (Linnaeus, 1767)

124. *Wachtliella stachydis* (Bremi, 1847)

125. *Zeuxidiplosis giardi* (Kieffer, 1896)

126. *Zygiobia carpini* (Löw, 1874).

#### ﻿Ceratopogonidae

The earliest records of these biting midges in Armenia are from volume 3 of the Catalogue of Palearctic Diptera (Soós and Papp 1988). The most recent papers to include a few records from Armenia are by Borkent and Dominiak (2020), El-Hawagry et al. (2020) and Przhiboro (2007).

1. *Atrichopogon albiscapula* Kieffer, 1918

2. *Atrichopogon appendiculatus* (Goetghebuer, 1920)

3. *Atrichopogon fulvipes* Remm, 1972

4. *Atrichopogon gillipaludosus* Remm, 1993

5. *Atrichopogon majusculus* Remm, 1961

6. *Atrichopogon meloesugans* Kieffer, 1922

7. *Bezzia albicornis* (Meigen, 1818)

8. *Bezzia nigrita* Clastrier, 1962

9. *Bezzia sevanica* Remm, 1974

10. *Bezzia tshernovskii* Remm, 1993

11. *Bezzia xanthocephala* Goetghebuer, 1911

12. *Brachypogon sevanicus* (Remm, 1974)

13. *Brachypogon vitiosus* (Winnertz, 1852)

14. *Ceratopogon caucasicus* Remm, 1967

15. *Ceratopogon crassinervis* (Goetghebuer, 1920)

16. *Ceratopogon niveipennis* Meigen, 1804

17. *Culicoides abchazicus* Dzhafarov, 1964

18. *Culicoides azerbajdzhanicus* Dzhafarov, 1962

19. *Culicoides bulbostylus* Khalaf, 1961

20. *Culicoides delta* Edwards, 1939

21. *Culicoides firuzae* Dzhafarov, 1958

22. *Culicoides longicollis* Glukhova, 1971

23. *Culicoides musajevi* Dzhafarov, 1961

24. *Culicoides saevanicus* Dzhafarov, 1960

25. *Culicoides salinarius* Kieffer, 1914

26. *Culicoides segnis* Campbell & Pelham-Clinton, 1960

27. *Culicoides sejfadinei* Dzhafarov, 1958

28. *Culicoides tauricus* Gutsevich, 1959

29. *Culicoides tugaicus* Dzhafarov, 1960

30. *Culicoides zhogolevi* Remm & Zhogolev, 1968

31. *Dasyhelea bifida* Zilahi-Sebess, 1936

32. *Dasyhelea calycata* Remm, 1972

33. *Dasyhelea obscura* (Winnertz, 1852)

34. *Dasyhelea serristernum* Remm, 1967

35. *Forcipomyia biskraensis* Kieffer, 1923

36. *Forcipomyia chrysolopha* Kieffer, 1911

37. *Forcipomyia crassipes* (Winnertz, 1852)

38. *Forcipomyia pulchrithorax* Edwards, 1924

39. *Forcipomyia sahariensis* Kieffer, 1923

40. *Leptoconops bezzii* (Noè, 1905)

41. *Leptoconops borealis* Gutsevich, 1945

42. *Leptoconops laurae* (Weiss, 1912)

43. *Leptoconops nigripes* Dzhafarov, 1961

44. *Monohelea spathulata* Remm, 1993

45. *Palpomyia aterrima* Goetghebuer, 1921

46. *Palpomyia flavipes* (Meigen, 1804)

47. *Palpomyia nigritarsalis* Remm, 1976

48. *Schizohelea leucopeza* (Meigen, 1804)

49. *Serromyia subinermis* Kieffer, 1919

#### ﻿Chironomidae

Records for this family are from the World Catalogue of Chironomidae parts 1 and 2 by Ashe and O’Connor (2009, 2012), with an additional record in Pont (2013a).

1. *Ablabesmyia phatta* (Egger, 1863)

2. *Boreoheptagyia legeri* (Goetghebuer, 1933)

3. *Orthocladius abiskoensis* Thienemann & Kruger, 1937

4. *Paratendipes nudisquama* (Edwards, 1929)

5. *Prodiamesa olivacea* (Meigen, 1818)

6. *Psectrocladius barbimanus* (Edwards, 1929)

7. *Tvetenia duscoloripes* (Goetghebuer, 1936)

#### ﻿Culicidae

This list of mosquito records is from the recent work of Shcherbakov et al. (2023).

1. *Aedes albopictus* (Skuse, 1894)

2. *Aedes annulipes* (Meigen, 1830)

3. *Aedes caspius* (Pallas, 1771)

4. *Aedes cataphylla* Dyar, 1916

5. *Aedes cinereus* Wiedemann, 1818

6. *Aedes dorsalis* (Meigen, 1830)

7. *Aedes flavescens* (Müller, 1764)

8. *Aedes geniculatus* (Olivier, 1791)

9. *Aedes punctor* (Kirby, 1837)

10. *Aedes vexans* (Meigen, 1830)

11. *Anopheles claviger* (Meigen, 1804)

12. *Anopheles hyrcanus* (Pallas, 1771)

13. *Anopheles maculipennis* Meigen, 1818

14. *Anopheles plumbeus* Stephens, 1828

15. *Anopheles sacharovi* Favre, 1903

16. *Anopheles superpictus* Grassi, 1899

17. *Coquillettidia richiardii* (Ficalbi, 1889)

18. *Culex hortensis* Ficalbi, 1889

19. *Culex martinii* Medschid, 1930

20. *Culex mimeticus* Noè, 1899

21. *Culex modestus* Ficalbi, 1889

22. *Culex pipiens* Linnaeus, 1758

23. *Culex territans* Walker, 1856

24. *Culex theileri* Edwards, 1912

25. *Culex torrentium* Martini, 1925

26. *Culiseta alaskaensis* (Ludlow, 1906)

27. *Culiseta annulata* (Schrank, 1776)

28. *Culiseta longiareolata* (Macquart, 1838)

29. *Culiseta morsitans* (Theobald, 1901)

30. *Culiseta subochrea* (Edwards, 1921)

31. *Uranotaenia unguiculata* Edwards, 1913

#### ﻿Dixidae

The family is listed from Oboňa et al. (2017).

1. *Dixa puberula* Loew, 1849

2. *Dixa submaculata* Edwards, 1920

3. *Dixella obscura* (Loew, 1849)

#### ﻿Keroplatidae

Armenian records for these three species have been found in three different papers (Joost and Plassmann 1985; Zaitzev 1994; Evenhuis 2006).

1. *Macrocera angulata* Meigen, 1818

2. *Macrocera crassicornis* Winnertz, 1864

3. *Macrocera lutea* Meigen, 1804

#### ﻿Limoniidae

The family is one of the well-studied families in Armenia (Oboňa et al. 2016; Hakobyan and Jenderedjian 2023).

1. *Achyrolimonia decemmaculata* (Loew, 1873)

2. *Afrolimnophila minima* (Savchenko, 1971)

3. *Antocha vitripennis* (Meigen, 1830)

4. *Dicranomyia caledonica* Edwards, 1926

5. *Dicranomyia circassica* Lackschewitz, 1941

6. *Dicranomyia didyma* (Meigen, 1804)

7. *Dicranomyia longipennis* (Schummel, 1829)

8. *Dicranomyia lucida* Meijere, 1918

9. *Dicranomyia melanantha* Savchenko, 1984

10. *Dicranomyia modesta* (Meigen, 1818)

11. *Dicranomyia pallidinota* Starý, 2009

12. *Dicranomyia pontica* Lackschewitz, 1941

13. *Dicranomyia transsilvanica* Lackschewitz, 1928

14. *Dicranoptycha livescens* Loew, 1870

15. *Ellipteroides omissus* (Lackschewitz, 1940)

16. *Erioconopa symplectoides* (Kuntze, 1914)

17. *Erioconopa trivialis* (Meigen, 1818)

18. *Erioptera bivittata* (Loew, 1873)

19. *Erioptera fusculenta* Edwards, 1938

20. *Erioptera lutea* Meigen, 1804

21. *Gonomyia basilobata* Alexander, 1975

22. *Gonomyia papposa* Savchenko, 1983

23. *Hexatoma gaedii* (Meigen, 1830)

24. *Hexatoma haiasana* (Savchenko, 1972)

25. *Hoplolabis amseliana* (Nielsen, 1961)

26. *Hoplolabis iranica* (Alexander, 1973)

27. *Hoplolabis yezoana* (Alexander, 1924)

28. *Idiocera laterospina* (Alexander, 1975)

29. *Ilisia maculata* (Meigen, 1804)

30. *Limnophila pictipennis* (Meigen, 1818)

31. *Limonia flavipes* (Fabricius, 1787)

32. *Limonia fusca* Meigen, 1804

33. *Limonia hercegovinae* (Strobl, 1898)

34. *Limonia macrostigma* (Schummel, 1829)

35. *Limonia nubeculosa* Meigen, 1804

36. *Limonia stigma* (Meigen, 1818)

37. *Limonia subaequalis* Savchenko, 1979

38. *Metalimnobia quadrinotata* (Meigen, 1818)

39. *Molophilus decoloratus* Savchenko, 1978

40. *Molophilus hebetatus* Savchenko, 1976

41. *Molophilus obscurus* (Meigen, 1818)

42. *Molophilus ochraceus* (Meigen, 1818)

43. *Molophilus pleuralis* Meijere, 1920

44. *Molophilus politonigrus* Savchenko, 1983

45. *Molophilus propinquus* (Egger, 1864)

46. *Molophilus urodontus* Savchenko, 1978

47. *Neolimnomyia rufulum* Savchenko, 1979

48. *Ormosia cuspidata* Savchenko, 1973

49. *Ormosia fascipennis* (Zetterstedt, 1838)

50. *Ormosia hederae* (Curtis, 1835)

51. *Ormosia longispina* Savchenko, 1983

52. *Paradelphomyia brevifurca* Savchenko, 1976

53. *Paradelphomyia fuscula* (Loew, 1873)

54. *Paradelphomyia senilis* (Haliday, 1833)

55. *Phylidorea ferruginea* (Meigen, 1818)

56. *Phyllolabis ghilarovi* Savchenko, 1983

57. *Pseudolimnophila melanura* Savchenko, 1984

58. *Pseudolimnophila sepium* (Verrall, 1886)

59. *Rhabdomastix eugeni* Stary, 2004

60. *Rhabdomastix filata* Starý, 2004

61. *Rhipidia maculata* Meigen, 1818

62. *Symplecta hybrida* (Meigen, 1804)

#### ﻿Mycetophilidae

Records of Armenian fungus gnats are are aslo very few (Joost and Plassmann 1985; Zaitzev 1994).

1. *Allodia retracta* Plassmann, 1976

2. *Exechiopsis pseudindecisa* (Laštovka & Matile, 1974)

3. *Leptomorphus quadrimaculatus* (Matsumura, 1916)

4. *Phronia biarcuata* (Becker, 1908)

5. *Sciophila limbatella* Zetterstedt, 1852

#### ﻿Pediciidae

Species of this family have been studied recently in Armenia (Oboňa et al. 2016; Hakobyan and Jenderedjian 2023; Oosterbroek 2025).

1. *Dicranota crassicauda* Tjeder, 1972

2. *Dicranota iranensis* (Alexander, 1975)

3. *Dicranota landrocki* Czižek, 1931

4. *Dicranota subtilis* Loew, 1870

5. *Pedicia occulta* (Meigen, 1830)

#### ﻿Psychodidae

Records of this important family of biting sand flies are from two papers (Perfil’ev 1937; Ježek et al. 2018).

1. *Clogmia albipunctata* (Williston, 1893)

2. *Joostiella caucasica* Vaillant, 1983

3. *Logima albipennis* (Zetterstedt, 1850)

4. *Parajungiella abchazica* Ježek, 1985

5. *Paramormia fratercula* (Eaton, 1893)

6. *Paramormia polyascoidea* ((Krek, 1971)

7. *Paramormia ustulata* (Haliday, 1856)

8. *Pericoma blandula* Eaton, 1893

9. *Pericoma bosniaca* Krek, 1966

10. *Pericoma exquisita* Eaton, 1893

11. *Pericoma platystyla* Wagner, 1986

12. *Peripsychoda auriculata* (Haliday, 1839)

13. *Phlebotomus alexandri* Sinton, 1928

14. *Phlebotomus chinensis* Newstead, 1916 [Not in the Jezek list]

15. *Phlebotomus cucasicus* Martsinovsky, 1917 [Correct spelling according to Jezek]

16. *Phlebotomus halepensis* Theodor, 1958

17. *Phlebotomus kandelakii* Shurenkova, 1929

18. *Phlebotomus papatasi* (Scopoli, 1786)

19. *Phlebotomus sergenti* Parrot, 1917

20. *Phlebotomus simici* Nitzulescu, 1931

21. *Phlebotomus similis* Perfil’ev, 1963 (sensu Artemiev & Neronov, 1984)

22. *Phlebotomus syriacus* Adler & Theodor, 1931

23. *Phlebotomus tobbi* Adler, Theodor & Lourie, 1930

24. *Pneumia canescens* (Meigen, 1804)

25. *Pneumia joosti* (Wagner, 1981)

26. *Pneumia nubila* (Meigen, 1818)

27. *Pneumia pilularia* (Tonnoir, 1940)

28. *Psychoda uniformata* Haseman, 1907

29. *Psychodocha cinerea* (Banks, 1894)

30. Saraiella ressli ssp. ressli Wagner, 1981

31. *Sergentomyia minuta* (Rondani, 1843)

32. *Sergentomyia palestinensis* (Adler & Theodor, 1927)

33. *Sergentomyia pawlowskyi* (Perfiliev, 1933)

34. *Thornburghiella veve* Oboňa &Ježek, 2017

35. *Tinearia alternata* (Say, 1824)

36. *Ulomyia cognata* (Eaton, 1893)

37. *Yomormia petrovi* Ježek,1985

#### ﻿Ptychopteridae

The records for this family are from Oboňa et al. (2017).

1. *Ptychoptera alina* Krzemiñski & Zwick, 1993

2. *Ptychoptera contaminata* (Linnaeus, 1758)

#### ﻿Scatopsidae

The records for this family are from Oboňa et al. (2017).

1. *Efcookella albitarsis* (Zetterstedt, 1850)

2. *Reichertella nigra* (Meigen, 1804)

3. *Swammerdamella brevicornis* (Meigen, 1830)

#### ﻿Simuliidae

Many species of biting black flies are of importance in the medical and veterinary fields, and they are also used in the evaluation of the water quality of Armenian rivers. The Armenian fauna has been investigated by Terterian (1968) and in several other works (Soós and Papp 1984; Adler and Werner (2010), summarised in the inventory of world species by Adler (2025).

1. *Greniera nairica* Terteryan, 1972

2. *Metacnephia hajotsdzorensis* (Terteryan, 1962)

3. *Metacnephia hirta* (Rubtsov & Terteryan, 1954)

4. *Metacnephia persica* (Rubtsov, 1940)

5. *Metacnephia subalpina* (Rubtsov, 1956)

6. *Metacnephia terterjani* (Rubtsov, 1955)

7. *Prosimulium tomosvaryi* (Enderlein, 1921)

8. *Prosimulium frontatum* Terteryan, 1956

9. *Prosimulium petrosum* Rubtsov, 1955

10. *Prosimulium rachiliense* Dzhafarov, 1954

11. *Simulium akopi* (Chubareva & Kachvoryan, 2000)

12. S*imulium alajense* Rubtsov, 1938

13. *Simulium alizadei* Dzhafarov, 1954

14. *Simulium angustipes* Edwards, 1915

15. *Simulium armeniacum* (Rubtsov, 1955)

16. *Simulium arpiense* (Terteryan & Kachvoryan, 1982)

17. *Simulium aureofulgens* Terteryan, 1949

18. *Simulium australe* (Rubtsov, 1955)

19. *Simulium azerbaidzhanicum* (Dzhafarov, 1953)

20. *Simulium bergi* Rubtsov, 1956

21. *Simulium bezzii* Corti, 1914

22. *Simulium brachyantherum* Rubtsov, 1947

23. *Simulium bukovskii* Rubtsov, 1940

24. *Simulium chubarevae* Kachvoryan & Terteryan, 1981

25. *Simulium cryophilum* (Rubtsov, 1959)

26. *Simulium debacli* Terteryan, 1952

27. *Simulium delizhanense* (Rubtsov, 1955)

28. *Simulium djafarovi* (Rubtsov, 1962)

29. *Simulium equinum* (Linnaeus, 1758)

30. *Simulium erythrocephalum* (De Geer, 1776)

31. *Simulium flaveolum* Rubtsov, 1940

32. *Simulium fontanum* Terteryan, 1952

33. *Simulium fontium* (Rubtsov, 1955)

34. *Simulium garniense* (Rubtsov, 1955)

35. *Simulium gviletense* (Rubtsov, 1956)

36. *Simulium hiemale* (Rubtsov, 1956)

37. *Simulium kiritshenkoi* (Rubtsov, 1940)

38. *Simulium kurense* Rubtsov & Dzhafarov, 1951

39. *Simulium litshkense* (Rubtsov, 1956)

40. *Simulium margaritae* (Rubtsov, 1956)

41. *Simulium maritimum* (Rubtsov, 1956)

42. *Simulium murvanidzei* (Rubtsov, 1955)

43. *Simulium nigrofusipes* Rubtsov, 1947

44. *Simulium noelleri* Friederichs, 1920

45. *Simulium ocreastylum* (Rubtsov, 1956)

46. *Simulium paraequinum* Puri, 1933

47. *Simulium paucicuspis* (Rubtsov, 1947)

48. *Simulium petricolum* (Rivosecchi, 1963)

49. *Simulium popowae* Rubtsov, 1940

50. *Simulium pseudequinum* Séguy, 1921

51. *Simulium reginae* Terteryan, 1949

52. *Simulium schamili* (Rubtsov, 1964)

53. *Simulium stenophallum* Terteryan, 1952

54. *Simulium tarnogradskii* Rubtsov, 1940

55. *Simulium turgaicum* Rubtsov, 1940

56. *Simulium variegatum* Meigen, 1818

57. *Simulium vernum* Macquart, 1826

58. *Simulium vitile* (Rubtsov, 1955)

59. *Simulium zakhariense* (Rubtsov, 1955)

60. *Sulcicnephia znoikoi* (Rubtsov, 1940)

#### ﻿Tipulidae

Records for this family are from Hakobyan and Jenderedjian (2023) and Catalogue of the Craneflies of the World (Oosterbroek 2025).

1. *Nephrotoma analis* (Schummel, 1833)

2. Nephrotoma cornicina ssp. cornicina (Linnaeus, 1758)

3. Nephrotoma croceiventris (Strobl, 1909) ssp. lindneri (Mannheims, 1951)

4. *Nephrotoma nox* (Riedel, 1910)

5. Nephrotoma pratensis ssp. pratensis (Linnaeus, 1758)

6. *Nephrotoma rossica* (Riedel, 1910)

7. *Nephrotoma scalaris* (Meigen, 1818)

8. *Prionocera subserricornis* (Zetterstedt, 1851)

9. *Tipula aurita* Riedel, 1920

10. *Tipula flavolineata* Meigen, 1804

11. *Tipula fulvipennis* De Geer, 1776

12. *Tipula irrequieta* Alexander, 1936

13. *Tipula lateralis* Meigen, 1804

14. *Tipula lunata* Linnaeus, 1758

15. *Tipula luteobasalis* Savchenko, 1964

16. *Tipula macropyga* Savchenko, 1952

17. *Tipula obscuriventris* Strobl, 1900

18. *Tipula orientalis* Lackschewitz, 1930

19. *Tipula osmana* Mannheims, 1963

20. *Tipula peliostigma* Schummel, 1833

21. *Tipula pullata* Savchenko, 1960

22. *Tipula saginata* Bergroth, 1891

23. *Tipula semivittata* Savchenko, 1960

24. *Tipula serrulifera* Alexander, 1942

25. *Tipula stigmatella* Schummel, 1833

26. *Tipula subcunctans* Alexander, 1921

27. *Tipula transcaucasica* Savchenko, 1961

28. *Tipula unca* Wiedemann, 1817

29. *Tipula variicornis* Schummel, 1833

30. *Tipula verrucosa* Pierre, 1920

31. *Tipula zaitzevi* Savchenko, 1952

## ﻿Discussion

The Checklist given above is a preliminary one which will provide future researchers with a base-line from which to analyze and add to the Diptera diversity of Armenia and the region. The Caucasus range as a whole remains poorly studied for Diptera. Only a few families of economic or ecological importance such as the Syrphidae, Muscidae and Drosophilidae have received adequate attention from entomologists (Mengual et al. 2020; Murvanidze et al. 2022; Pont and Parchami-Araghi 2024). There are several other families which may also occur in Armenia but as we have not found any published records they are not included in this Checklist. One of the most important families which is not included here is the Syrphidae which is estimated to have 300 species in Armenia (Grigoryan et al., unpublished data). Of the species listed above in the Checklist, only two are listed in the Red Book of Armenia, both from the family Asilidae: *Machimus erevanensis* Richter, 1963, and *Satanas gigas* Eversmann, 1855 (Red Book 2010).

Several families’ containing pest species such as the Agromyzidae, Chloropidae, Drosophilidae, Ephydridae, Opomyzidae, Piopilidae, Tephritidae may also be considerably more speciose in Armenia as the records given here are from relatively old sources (Avetyan and Mirzoyan 1976; Mirzoyan 1977). Based on the Checklist, the most diverse family in Armenia is the Bombyliidae with 253 recorded species, followed by the Muscidae with 208 records. Families represented by single species were more common: Anthomyzidae, Curtonotidae, Lauxaniidae, Micropezidae, Phaeomyiidae, Piophilidae, Rhinophoridae, Blephariceridae. Of the families of Diptera listed in the "*Systema Dipterorum*" (Evenhuis and Pape 2025), only 53 have been found in Armenia. However, it is very likely that further families will be found. We noted that in many families (Agromyzidae, Asilidae, Bombyliidae, Chironomidae, Culicidae, Drosophilidae, Ephydridae, Lauxaniidae, Micropezidae, Muscidae, Otitidae, Psilidae, Pyrgotidae, Sarcophagidae, Sciomyzidae, Simuliidae, Syrphidae, Tabanidae, Tachinidae, Ulidiidae) published references gave the species distribution area as “Transcaucasus” or “Caucasus”, which we have considered to be too general and have not included in the Checklist.

It has been estimated that the Armenian Diptera fauna should contain approximately 2000 species (CBD 2014), and we confidently expect that future research will lead to the discovery of additional families and species of Armenian Diptera.

## ﻿Additional information

### Use of AI

No use of AI was reported.

### Author ORCIDs

Meri Arzumanyan https://orcid.org/0000-0002-9777-7830

Natela Tarzyan https://orcid.org/0009-0002-8468-5766

Björn Rulik https://orcid.org/0000-0002-2808-9305

Adrian Pont https://orcid.org/0000-0003-4541-9182

### Data availability

All of the data that support the findings of this study are available in the main text or Supplementary Information.

## Supplementary materials

Supplementary material 1 Diptera species list from references Data type xlsx Explanation note This file contains species list extracted from references with their distribution, synonyms and other taxomomic information. https://binary.pensoft.net/file/1487118This dataset is made available under the Open Database License (http://opendatacommons.org/licenses/odbl/1.0/). The Open Database License (ODbL) is a license agreement intended to allow users to freely share, modify, and use this Dataset while maintaining this same freedom for others, provided that the original source and author(s) are credited.Meri Arzumanyan, Natela Tarzyan, Björn Rulik, Adrian Pont

Supplementary material 2 New records of Diptera with distribution and some collection data Data type xlsx Explanation note List contains 106 Diptera species new records for Armenia, with their distribution and collection data. https://binary.pensoft.net/file/1487119This dataset is made available under the Open Database License (http://opendatacommons.org/licenses/odbl/1.0/). The Open Database License (ODbL) is a license agreement intended to allow users to freely share, modify, and use this Dataset while maintaining this same freedom for others, provided that the original source and author(s) are credited.Meri Arzumanyan, Natela Tarzyan, Björn Rulik, Adrian Pont
